# Hypoxia-induced transcriptional stress is mediated by ROS-induced R-loops

**DOI:** 10.1093/nar/gkad858

**Published:** 2023-10-16

**Authors:** Tiffany S Ma, Katja R Worth, Conor Maher, Natalie Ng, Chiara Beghè, Natalia Gromak, Anna M Rose, Ester M Hammond

**Affiliations:** Department of Oncology, University of Oxford, Oxford OX3 7DQ, UK; Department of Oncology, University of Oxford, Oxford OX3 7DQ, UK; Department of Oncology, University of Oxford, Oxford OX3 7DQ, UK; Department of Oncology, University of Oxford, Oxford OX3 7DQ, UK; Sir William Dunn School of Pathology, University of Oxford, Oxford OX1 3RE, UK; Sir William Dunn School of Pathology, University of Oxford, Oxford OX1 3RE, UK; Department of Pediatrics, University of Oxford, Oxford OX3 9DU, UK; Department of Oncology, University of Oxford, Oxford OX3 7DQ, UK

## Abstract

Hypoxia is a common feature of solid tumors and is associated with poor patient prognosis, therapy resistance and metastasis. Radiobiological hypoxia (<0.1% O_2_) is one of the few physiologically relevant stresses that activates both the replication stress/DNA damage response and the unfolded protein response. Recently, we found that hypoxia also leads to the robust accumulation of R-loops, which led us to question here both the mechanism and consequence of hypoxia-induced R-loops. Interestingly, we found that the mechanism of R-loop accumulation in hypoxia is dependent on non-DNA damaging levels of reactive oxygen species. We show that hypoxia-induced R-loops play a critical role in the transcriptional stress response, evidenced by the repression of ribosomal RNA synthesis and the translocation of nucleolin from the nucleolus into the nucleoplasm. Upon depletion of R-loops, we observed a rescue of both rRNA transcription and nucleolin translocation in hypoxia. Mechanistically, R-loops accumulate on the rDNA in hypoxia and promote the deposition of heterochromatic H3K9me2 which leads to the inhibition of Pol I-mediated transcription of rRNA. These data highlight a novel mechanistic insight into the hypoxia-induced transcriptional stress response through the ROS–R-loop–H3K9me2 axis. Overall, this study highlights the contribution of transcriptional stress to hypoxia-mediated tumorigenesis.

## Graphical Abstract

**Graphical Abstract:**
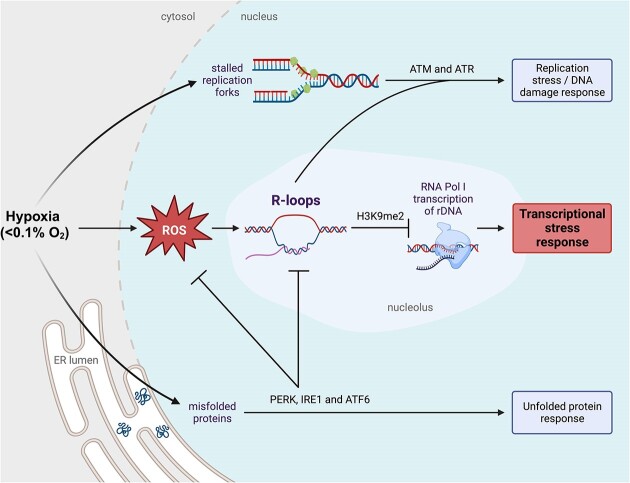

## Introduction

Most solid tumors contain regions of low oxygen (hypoxia) that significantly reduce the effectiveness of cancer therapy. Clinical studies across multiple tumor types have established that hypoxia is associated with poor patient prognosis and a higher likelihood of metastasis. The hypoxic tumor microenvironment is characterized by elevated genomic instability, resistance to radiotherapy and chemotherapy, local immune suppression and selection for apoptosis-resistant sub-populations (1–3). A gradient of oxygen tensions occurs throughout tumors, reaching near anoxia in areas adjacent to necrotic regions. Whereas a range of low oxygen tensions leads to stabilization of the transcription factor, HIF-1α, radiobiological hypoxia (<0.1% O_2_) leads to a unique stress response including the unfolded protein response (UPR), and a replication stress-induced DNA damage response (DDR) (4–6). The hypoxia induced UPR downregulates protein synthesis and increases folding capacity of chaperones to cope with misfolded proteins that accumulate in the endoplasmic reticulum. The activated UPR includes signaling through three arms, initiated by PERK, IRE1 and ATF6 (6–8). Notably, the hypoxia-induced DDR occurs in the absence of detectable DNA damage (single or double strand breaks SSB/DSB) and also includes the accumulation of R-loops (9–12).

R-loops are three-stranded nucleic acid structures, comprised of an RNA:DNA hybrid and ssDNA, which have been recognized as accumulating during transcriptional stress. R-loops are frequently formed during transcription across many regions of the genome and act to regulate gene expression, however their dysregulation is associated with human diseases (13,14). Importantly, aberrant unresolved R-loops contribute to genomic instability by acting as a blockage to the replication machinery, leading to transcription-replication conflicts (TRCs) (13,15–17). Furthermore, exposed ssDNA in R-loops becomes accessible to mutagenic agents including cytidine deaminases (18). Previously, we described the accumulation of R-loops in hypoxia and found that the RNA:DNA helicase, senataxin (SETX), is induced as a result of UPR-mediated signaling, specifically through PERK/ATF4 (10). Although R-loops can lead to or result from DNA damage in response to other stresses, hypoxia increases R-loop levels in the absence of DNA damage (10). The mechanism of R-loop accumulation and their function in hypoxia remain unclear.

A less characterized response to hypoxia is the repression of transcription rates or transcriptional stress. Transcriptional stress can be broadly defined as the global repression of RNA synthesis and can occur as a result of the transcription machinery encountering obstacles in the form of DNA damage, topological constraints, stalled replication forks from cellular stresses such as heat shock, oxidative stress, nutrient deprivation or transcription inhibitors (19–22). Although hypoxia is known to lead to reduced transcription rates, few studies exploring this mechanism have been carried out and they mainly focus on mechanisms repressing RNA Polymerase II (Pol II) transcribed genes through transcriptional repressor proteins (23,24). However, approximately 70% of cellular transcription is attributed to RNA polymerase I (Pol I) mediated transcription of the ribosomal DNA (rDNA) located in the nucleolus (25–29). To our knowledge, the mechanism underlying Pol I-mediated transcriptional repression has not been investigated previously in conditions of radiobiological hypoxia (<0.1% O_2_) although global transcriptional repression has been described (30). An additional hallmark of transcriptional stress is reorganization of the nucleolus, including changes in the distribution of proteins between the nucleolus, nucleoplasm and cytoplasm (31).

Here, we confirmed that Pol I-mediated transcription is reduced in radiobiological hypoxia (<0.1% O_2_), and this is accompanied by nucleolar reorganization. In these same conditions, R-loops accumulate in the nucleolus in a reactive oxygen species (ROS)-dependent manner. Specifically, hypoxia-induced ROS cause R-loop accumulation at the rDNA leading to an increase in the heterochromatic mark, H3K9me2, and reduced Pol I-mediated transcription. Together, these data suggest that not only are R-loops a marker of transcriptional stress, but they also contribute to hypoxia-induced transcriptional stress by repressing Pol I transcription and causing nucleolar reorganization.

## Materials and methods

### Cell lines and reagents

Cells were grown in DMEM supplemented with 10% FBS, in a standard humidified incubator at 37°C and 5% CO_2_. Cell lines include A549 (human lung adenocarcinoma, ATCC), HCT116 (human colorectal cancer cells, a gift from Prof. Bert Vogelstein, Johns Hopkins School of Medicine), RKO (human colorectal cancer cells, ATCC), MRC5 (human lung fibroblast cells, from Prof. Geoff Higgins, University of Oxford), HCT116^ATR/-flox^ (32,33), HCT116 and HCT116*^HIF1-a-/−^* (34). Cells were verified mycoplasma free at regular intervals using a HEK-Blue™ detection kit (Invivogen). JetPrime (Polyplus transfection) was used for plasmid transfections. Plasmids used include ppyCAG_RNaseH1_WT (Addgene #111906), ppyCAG_RNaseH1_D210N (Addgene #111904), ppyCAG_RNaseH1_WKKD (Addgene #111905) and GFP-tagged RNase H1^WT^ (Prof. Natalia Gromak, University of Oxford).

### Hypoxia

Hypoxia treatments were carried out in a Bactron II, Bactronez-2 anaerobic chamber (Shel lab) or a Don Whitley Scientific H35 chamber. For experiments at <0.1% O_2_ cells were plated on glass dishes. Cells were harvested inside the chamber with equilibrated solutions.

### Total ROS measurement by CellRox

During the last 10 min of treatment, cells were incubated in the dark at 37°C with media containing CellRox Green (5 μM) (Invitrogen). Cells were fixed and analyzed immediately. Data acquisition was carried out for 10 000 cells per experimental condition using the CytoFLEX flow cytometer (Beckman Coulter) and CytExpert Software. Analysis after acquisition was carried out using the FlowJo software (BD Biosciences).

### H_2_O_2_ measurement by pHyper-nuc

Cells grown on coverslips were transfected 16–18 h before treatment with the pHyper-Nuc plasmid (Evrogen #FP944). After treatment, cells were treated and fixed with 4% PFA. Coverslips were mounted with DAPI mounting media and visualized on a LSM780 confocal microscope (Carl Zeiss Microscopy Ltd), using a 63×/1.40 Oil DIC M27 Plan-ApoChromat objective lens. DAPI was excited with laser line 405 nm and emission collected between 410 and 495 nm. The fluorescent pHyper-Nuc was excited with laser line 488 nm and emission collected between 495 and 596 nm. Nuclear intensity of fluorescence was quantified using ImageJ software.

### EU staining

5′ethynyl uridine (EU) (0.5 mM) (Jena Bioscience) was added to cells grown on coverslips for the last hour of treatment. Nascent transcription was measured using Click-iT Alexa Fluor 647 labeling kit (Thermo Fisher). Cells were visualized on a LSM710 confocal microscope (Carl Zeiss Microscopy Ltd). Nuclear intensity was determined using the ImageJ software.

### RT-qPCR

RNA was prepared using TRIzol (Invitrogen/Life Technologies). For RT-qPCR expression analysis cDNA was reverse transcribed from total RNA using the Verso kit (Thermo Scientific). qPCR was performed using SYBR Green PCR Master Mix kit (Applied Biosystems) in a 7500 FAST Real-Time PCR thermocycler with v2.0.5 software (Applied Biosystems). RNA fold change was calculated using a 2^–ΔΔCt^ method in relation to the 18S reference gene. The mean of three biological replicates ± SEM is shown. All primer sequences available in the supplementary information (Supplementary Table S1).

### Western blotting

Cells were lysed in UTB (9 M urea, 75 mM Tris–HCl pH 7.5, 0.15 M β-mercaptoethanol) and briefly sonicated. Proteins were separated on a 4–20% polyacrylamide gel (Bio-Rad) and transferred onto a nitrocellulose membrane (Bio-Rad). Odyssey IR imaging technology (LI-COR Biosciences) was used for imaging. Antibodies used; Abcam: H3 (ab1791), nucleolin (ab22758); BD Biosciences: GRP78 (610979), HIF-1α (610958); Bethyl: KAP1 (A300-274A); Cell Signaling: CHK1-S345 (2341), G9a (3306), H3 (14269), H3K9me2 (4658), KAP-S824 (4127), p53-S15 (9284), PERK (3192); Invitrogen: V5 (R960-25); Millipore: γ-H2AX (05–636), H2AX (07–627), H3K9me3 (07–523); Santa Cruz: β-actin (AC-15), ATR (sc-515173), CHK1 (sc-8408), p53 (sc-126), RNase H1 (sc-365267).

### Immunofluorescence and microscopy

Cells grown on coverslips were fixed with 4% PFA. Cells were permeabilized with 0.1% Triton X-100 and blocked in 2% BSA PBS. Antibodies used; Abcam: 8-oxoguanine (ab206461), fibrillarin (ab4566), nucleolin (ab22758); Invitrogen: V5 (R960-25); V5 (MA5-32053); Kerafast: S9.6 (Kf-Ab01137-23.0); Novus Biologicals: 53BP1 (NB100-304). Coverslips were mounted with DAPI mounting media (Invitrogen, P36962) and visualized on a LSM780 confocal microscope (Carl Zeiss Microscopy Ltd), using a 63×/1.40 Oil DIC M27 Plan-ApoChromat objective lens. DAPI was excited with laser line 405 nm and emission collected between 410 nm and 495 nm. Alexa Fluor 488 was excited with laser line 488 nm and emission collected between 495 nm and 596 nm. Alexa Fluor 594 was excited with laser line 594 nm and emission collected between 599 and 734 nm. Alexa Fluor 647 was excited with laser line 633 nm and emission collected between 638 and 755 nm. To determine the localization of nucleolin, samples were blinded and scored as either nucleolar or nucleoplasmic by at least two independent researchers. Quantification of microscopy for determining number of foci or nuclear intensities was carried out using FIJI/ImageJ software.

### R-loop detection by RNase H1^D210N^ mutant

Cells grown on coverslips were transfected with V5-tagged RNase H1^D210N^ (Addgene #111904) 16–20 h prior to treatment. Cells were incubated with ice-cold 0.2% Triton-X-100 for 2 min for pre-extraction before being fixed with 4% PFA. Immunofluorescence staining was carried out and coverslips were visualized on a LSM780 confocal microscope (Carl Zeiss Microscopy Ltd), using a 63×/1.40 Oil DIC M27 Plan-ApoChromat objective lens, as described in method for Immunofluorescence and microscopy. Nuclear intensity of V5-tagged RNase H1^D210N^ was determined using ImageJ software as a readout for R-loop levels.

### R-loop detection by S9.6 staining

Cells plated on coverslips were fixed in methanol at 4°C for 10 min as previously described (35). Coverslips were washed three times in PBS. RNase H treatment was carried out according to the manufacturers protocol (NEB) for 36 h. The cells were then washed in PBS, blocked in 2% BSA overnight. Primary antibodies (S9.6 (Kerafast) and nucleolin (Abcam)) were added and incubated at 4°C overnight. The coverslips were then washed three times and incubated with secondary antibody for 1 h at RT. Coverslips were mounted with DAPI mounting media and visualized on a LSM780 confocal microscope (Carl Zeiss Microscopy Ltd), using a 63×/1.40 Oil DIC M27 Plan-ApoChromat objective lens, as described in method for immunofluorescence and microscopy.

### DNA/RNA immunoprecipitation (DRIP)

DRIPs were carried out as previously described with minor modifications (35,36). Briefly, isolated cell nuclei were lysed in nuclear lysis buffer (50 mM Tris–HCl pH 8.0, 5 mM EDTA, 1% SDS) and digested for 3–5 h at 55°C with 40 μg of proteinase K (Sigma Aldrich). Genomic DNA was precipitated and resuspended in IP dilution buffer (16.7 mM Tris–HCl pH 8.0, 1.2 mM EDTA, 167 mM NaCl, 0.01% SDS, 1.1% Triton X-100). DNA was sonicated (Diagenode Bioruptor) and precleared with BSA-blocked protein A Dynabeads® (Invitrogen). Appropriate sonication was confirmed by running DNA samples on a 1.5% agarose gel. RNase H treatment was used as described by the manufacturer (M0297; NEB) for 2.5 h at 37°C for control samples. Immunoprecipitation was carried out by incubating equal concentrations of 15 μg genomic DNA with either S9.6 antibody (Kerafast) or beads only control overnight at 4°C on a rotating wheel. DNA was eluted with elution buffer (100 mM NaHCO3, 1% SDS) at RT for 30 min on a rotating wheel and treated with proteinase K (Thermofisher). DNA purification was carried out using the QIAquick PCR purification kit (Qiagen). Analysis was carried out using qPCR. The amount of immunoprecipitated material at a particular gene region was calculated as the percentage of input after subtracting the background signal (no antibody control). The values were then normalized to the normoxic untreated condition of the promoter primer. DRIP-qPCR primer sequences are available in Supplementary Table S1.

### Chromatin immunoprecipitation (ChIP)

Cells were fixed with 1% formaldehyde and quenched with 125 mM glycine. Cells were lysed with SDS lysis buffer (0.5% SDS, 10 mM EDTA, 50 mM Tris pH 8.1). After sonication, chromatin was precleared with protein A Dynabeads (Invitrogen) and immunoprecipitated with antibodies overnight at 4°C. The antibodies used were H3 (Abcam), H3K9me2 (Cell Signaling) and H3K9me3 (Abcam). The immune-antigen complexes were pulled down with Dynabeads beads and washed with a series of buffers – low salt wash buffer (0.1% SDS, 1% Triton X-100, 2 mM EDTA, 20 mM Tris pH 8.1, 150 mM NaCl), high salt wash (0.1% SDS, 1% Triton X-100, 2 mM EDTA, 20 mM Tris pH 8.1, 500 mM NaCl), LiCl wash buffer (1% Igepal, 10 mM Tris pH 8.1, 250 mM LiCl, 1 mM EDTA, 1% sodium deoxycholate), TE wash buffer (10 mM Tris pH 8.0, 1 mM EDTA). Eluted DNA was then treated with 40 μg of proteinase K for 1 h at 45°C. DNA purification was carried out and qPCR was carried out using the primers in Supplementary Table S1. The amount of immunoprecipitated material at a particular gene region was calculated as the percentage of input after subtracting the background signal (no antibody control).

### Gene pathway enrichment analysis

The ‘Similar Genes Detection’ tool in the Gepia2 platform was used to identify the top 100 genes that have the most similar expression pattern to SETX in patient samples from lung squamous cell carcinoma and lung adenocarcinoma (LUAD, LUSC) in the Cancer Genome Atlas Program (TCGA) datasets (37,38). The 100 genes were analyzed using gene ontology biological pathway analysis from the GeneCodis platform, resulting in the most significantly enriched pathways (39).

### Statistical analysis

The two-tailed, unpaired Student's *t*-test was used when comparing two means. * *P* ≤ 0.05, ** *P* ≤ 0.01, *** *P* ≤ 0.001, ****P ≤ 0.0001, ns (non-significant) *P* > 0.05. Errors bars represent mean ± standard error of the mean (SEM). The statistical test used is specified in each figure legend.

## Results

### R-loops accumulate in the nucleolus of hypoxic cells

**Figure 1.**
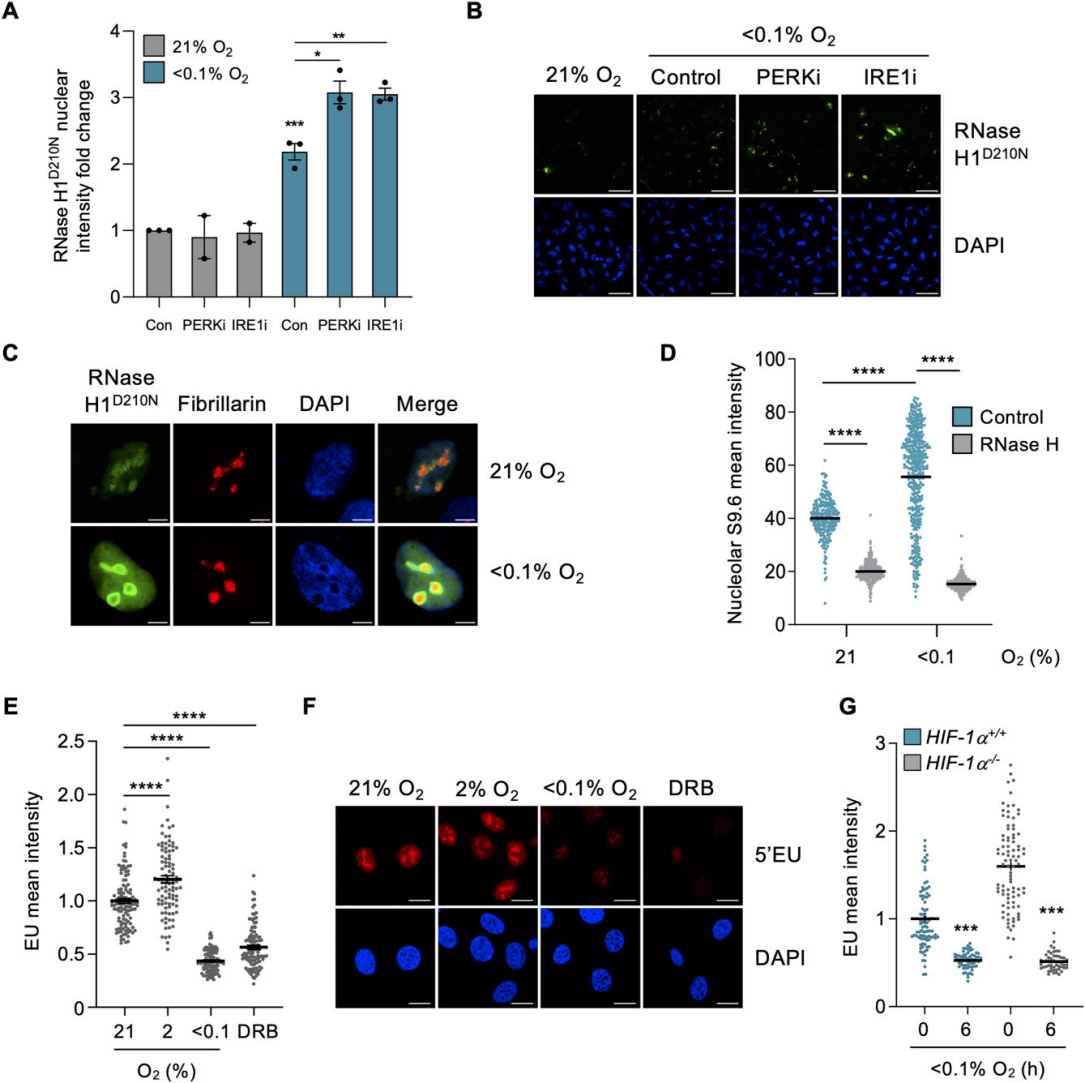
R-loops accumulate in the nucleoli of hypoxic cells despite reduced transcription rates. (**A**) A549 cells were transfected with V5-tagged RNase H1^D210N^ and exposed to 21 or <0.1% O_2_ (6 h) with or without a UPR inhibitor: PERK inhibitor (AMG PERK 44, 20 μM) or IRE1α inhibitor (4μ8c, 20 μM). Staining for V5 was carried out and nuclear intensity determined. Each data point represents the average from one of three biological repeats. (**B**) Representative images from part A. Scale bar represents 50 μM. (**C**) A549 cells were transfected with V5-tagged RNase H1^D210N^ and exposed to 21 or <0.1% O_2_ (6 h). Cells were fixed and co-stained for V5 (green), the nucleolar marker fibrillarin (red) and DAPI (blue). Scale bar represents 5 μM. *n*= 1 Images of a field of view shown in Supplementary Figure S1E. (**D**) HCT116 cells were exposed to 21 or <0.1% O_2_ (1.5 h), fixed and co-stained for nucleolin and R-loops using the S9.6 antibody. Where indicated, coverslips were treated with recombinant RNase H. The mean nucleolar intensity of S9.6 staining per cell was determined. Representative images are shown in Supplementary Figure S1F. (**E**) HCT116 cells were exposed to 21, 2 or <0.1% O_2_ (6 h) and labeled with 5′EU (0.5 mM). The mean nuclear intensity of 5′EU per cell was quantified. DRB (100 μM, 6 h), a global transcriptional inhibitor, was used as a control. (**F**) Representative images from part E. Scale bar represents 10 μM. (**G**) HCT116 and HCT116^HIF-1α-/-^ cells were exposed to 21 or <0.1% O_2_ (6 h) and labeled with 5′EU (0.5 mM). The mean nuclear intensity of 5′EU per cell was quantified. HIF-1α knockout was confirmed in Supplementary Figure S1I. (A–G) Data from three independent experiments (*n*= 3), mean ± standard error of the mean (SEM) are displayed unless otherwise indicated. * *P* < 0.05, ** *P* < 0.01, *** *P* < 0.001, **** *P* < 0.0001, ns (non-significant) *P* > 0.05. Unless otherwise indicated statistical significance refers to comparison to the normoxic control. In parts (D, E and G), each dot represents a cell. A minimum of 100 cells was imaged per condition in all microscopy experiments. The two-tailed, unpaired Student's *t*-test was used in parts (A, D, E and G).

We have shown previously that R-loops accumulate in hypoxic conditions and that levels increase further if the PERK arm of the UPR is inhibited (10). This observation is further supported here by the finding that thapsigargin (Thaps) and tunicamycin (Tuni), both of which, like hypoxia, activate the UPR also lead to R-loop accumulation (Supplementary Figure S1A, B) (40,41). Using the RNase H1 catalytic mutant (D210N) we confirmed that R-loop levels increase in hypoxia and extended our findings to demonstrate that both the PERK and IRE1 branches of the UPR act to restrict R-loop accumulation in hypoxia (Figure 1A, B). Markers of an active UPR including phosphorylation of PERK and accumulation of the spliced form of XBP-1 (XBP-1s) were induced in response to hypoxia and were reduced in response to AMG PERK 44 and 4μ8c respectively (Supplementary Figure S1C, D). The R-loops which accumulated in hypoxia appeared to be primarily localized to the nucleolus. To investigate this, we co-stained for RNase H1^D210N^ and a nucleolar marker, fibrillarin, and found an overlap (Figure 1C and Supplementary Figure S1E). In addition, we visualized R-loops using an orthogonal assay (S9.6 staining) combined with staining for the nucleolar marker, nucleolin, which has been identified as an R-loop binding protein (Figure 1D, images shown in Supplementary Figure S1F) (42). Notably, the accumulation of R-loops in the nucleolus, the major site of transcription in the cell, occurs in the context of reduced global transcription rates in hypoxic conditions (23). Here, we confirmed that global transcription, measured by EU incorporation, decreased in response to exposure to radiobiological hypoxia (<0.1% O_2_) but not milder hypoxia (2% O_2_) and that this occurs in both cancer (HCT116, RKO) and non-cancer cells (MRC5) (Figure 1E, F and S1G, H). 5,6-dichloro-1-beta-d-ribofuranosylbenzimidazole (DRB), a widely used transcription inhibitor, was used as a positive control, and found to reduce EU incorporation (43,44). Furthermore, we confirmed that this transcriptional repression occurs in a HIF-1α independent manner (Figure 1G, verification of HIF-1α status Supplementary Figure S1I).

### Hypoxia (<0.1% O_2_) induces transcriptional stress

**Figure 2.**
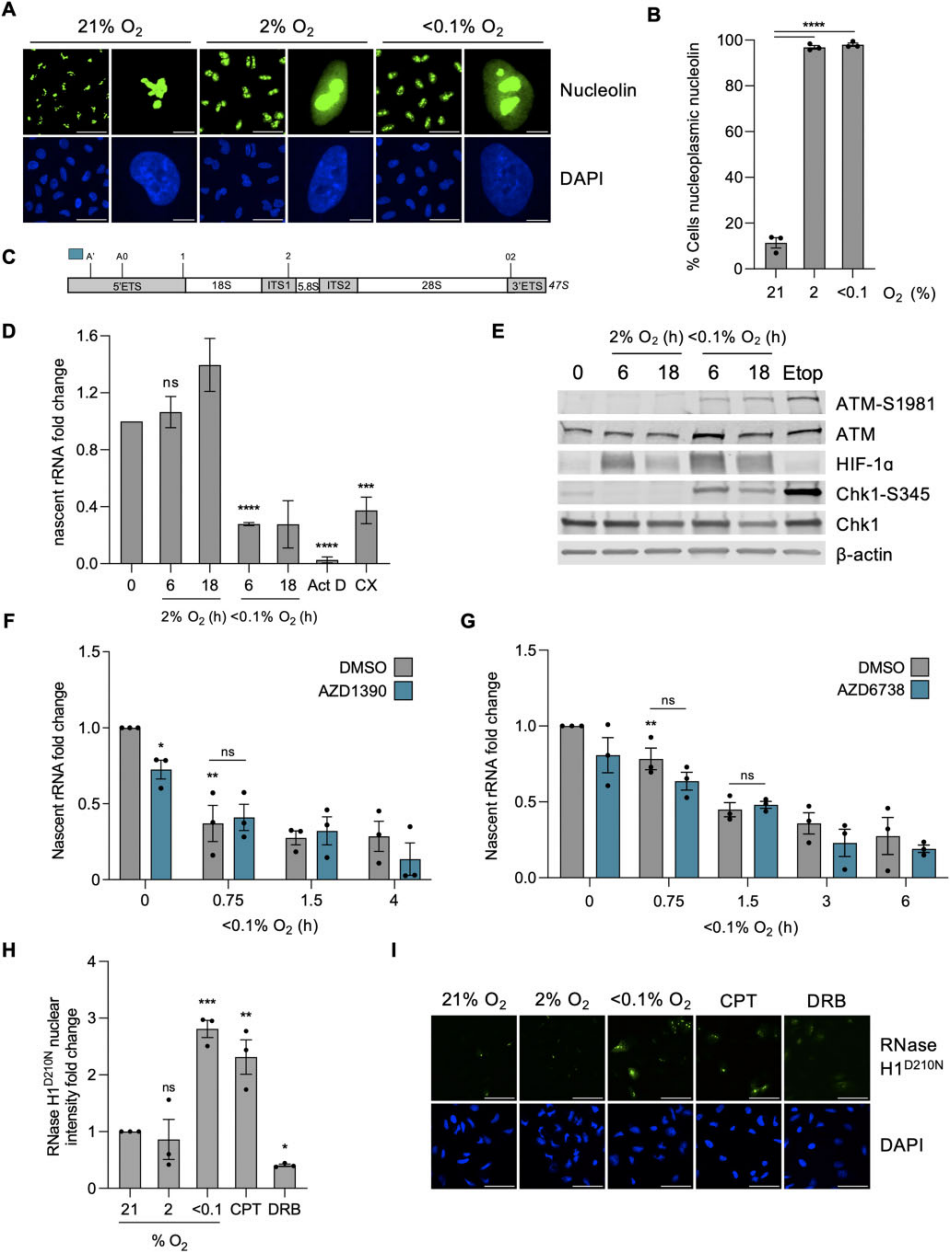
Hypoxia (<0.1% O_2_) leads to transcriptional stress independently of the DDR (**A**) A549 cells were exposed to 21, 2 or <0.1% O_2_ (6 h), fixed and stained for nucleolin (green) and DAPI (blue). Scale bar in field of view represents 50 μM. Scale bar in enlarged cell represents 5 μM. (**B**) Quantification of the percentage of cells with nucleoplasmic nucleolin from part (A). (**C**) Schematic of the initial product of RNPI transcription, 47S rRNA precursor. Initial cleavages occur at the A’ site in the 5′ETS (5′ externally transcribed spacer) region and at 02 site in the 3′ETS (3′ externally transcribed spacer) region before further cleavage (at sites A0, 1 and 2) and processing of the transcript, generating the mature 18, 28 and 5.8S. Blue bar indicates the amplicon from primers used to measure nascent 47S rRNA precursor levels, used subsequently in parts (D), (F) and (G). (**D**) HCT116 cells were exposed to 21, 2 or <0.1% O_2_ for the times indicated followed by RT-qPCR for the nascent 47S rRNA precursor. The rDNA transcription inhibitors Actinomycin D (Act D, 40 nM, 6 h) and CX5461 (CX, 100 nM, 6 h) were used as controls. (**E**) HCT116 cells were exposed to 21, 2 or <0.1% O_2_, for the times indicated, or etoposide (Etop) (25 μM, 6 h) followed by western blotting. β-Actin was used as a loading control. (**F**) HCT116 cells were pre-treated with ATM inhibitor AZD1390 (10 μM, 1 h) before being exposed to 21 or <0.1% O_2_ for the times indicated. RT-qPCR for the nascent 47S rRNA precursor is shown. ATM inhibition is confirmed in Supplementary Figure S2E. (**G**) HCT116 cells were pre-treated with ATM inhibitor AZD6738 (1 nM, 1 h) before being exposed to 21 or <0.1% O_2_ for the times indicated. RT-qPCR for the nascent 47S rRNA precursor is shown. ATM inhibition is confirmed in Supplementary Figure S2H. (**H**) A549 cells were transfected with V5-tagged RNase H1^D210N^ and exposed to 21, 2 or <0.1% O_2_ (6 h). CPT (10 μM, 1 h) and DRB (100 μM, 1 h) were used as controls to increase and decrease R-loop levels, respectively. Staining for V5 was carried out and the nuclear intensity was determined. (**I**) Representative images for the data shown in part H. V5 (green) and DAPI (blue) are shown. Scale bar represents 50 μM. (A–I**)** Data from three independent experiments (*n*= 3), mean ± standard error of the mean (SEM) are displayed unless otherwise indicated. * *P* < 0.05, ** *P* < 0.01, *** *P* < 0.001, **** *P* < 0.0001, ns (non-significant) *P* > 0.05. Unless otherwise indicated statistical significance refers to comparison to the normoxic control. In parts (B), (D), (F)–(H), each data point represents the average from one of three biological repeats, normalized to the untreated sample. A minimum of 100 cells was imaged per condition in all microscopy experiments. The two-tailed, unpaired Student's *t*-test was used in parts (B), (D), (F)–(H).

Agents causing transcriptional stress have been shown to cause translocation of nucleolin from the nucleolus into the nucleoplasm (45,46). Indeed, we observed a clear translocation of nucleolin from the nucleolus into the nucleoplasm in response to hypoxia, in both radiobiological levels of hypoxia (<0.1% O_2_) and mild levels of hypoxia (2% O_2_) (Figure 2A, B). We confirmed that the hypoxia-mediated changes in nucleolin were not due to changes in protein expression levels (Supplementary Figure S2A). As the nucleolus is the site of Pol I-mediated transcription, we verified that transcription of the rDNA was also reduced in hypoxic conditions, consistent with the pattern of global transcription repression. The product of Pol I transcription, 47S rRNA transcript, is initially cleaved at site A’ in the 5′ETS region before further cleavage and processing of the transcript occurs to generate the mature 18, 28 and 5.8S rRNAs (shown schematically in Figure 2C) (28,47). Using primers to the 5′ETS region, the relative levels of nascent unprocessed 47S rRNA precursor were determined and confirmed that rDNA transcription was repressed in <0.1% but not at 2% O_2_, consistent with the EU incorporation shown previously in both A549 and HCT116 cells (Figures 2D, S2B). As expected, 47S levels were also significantly reduced by the Pol I inhibitors, Actinomycin D, CX5461 and BMH-21. To further verify our findings, we carried out northern blotting to validate the hypoxia-mediated impact on rDNA transcription determined by RT-qPCR. Northern blotting showed a reduction in transcription of the 47S in hypoxia. Interestingly, after returning cells to normoxic conditions (reoxygenation) for just 1-h, basal levels of transcription were restored (Supplementary Figure S2C, D).

The role of ATM/ATR in repressing Pol I activity has been described as dependent on the presence of DSBs, which do not accumulate in hypoxic conditions (11,48,49). However, as the DDR is activated with similar kinetics to the repression of rDNA transcription in hypoxia, we asked if ATM/ATR could also play a role in hypoxia (Figure 2E). As expected, treatment with an ATM inhibitor (AZD1390) reduced markers of the DDR (ATM-S1981 and KAP1-S824) in hypoxia (Supplementary Figure S2E). Interestingly, treatment with AZD1390 did not rescue the repression of 47S levels suggesting that transcriptional repression of rDNA at <0.1% O_2_ occurs in an ATM independent manner (Figure 2F). Supportively, treatment with an alternative ATM inhibitor (KU55933) also failed to alleviate transcriptional repression of the rDNA in <0.1% O_2_ (Supplementary Figure S2F, G). Similarly, to investigate a potential role of hypoxia-induced ATR activity in the repression of rDNA transcription, cells were treated with an ATR inhibitor (AZD6738). ATR activity increased in response to hypoxia as evidenced by increased phosphorylation of Chk1 which was abrogated by AZD6738 (Supplementary Figure S2H). As seen after ATM inhibition, 47S repression in <0.1% O_2_ was unaffected by ATR inhibition (Figure 2G). To further support this finding, we used a HCT116*^ATR/-flox^* cell line containing one intact copy of the *ATR* allele which has significantly reduced levels of endogenous ATR compared to the wild-type cell line (Supplementary Figure S2I). HCT116*^ATR/-flox^* cells demonstrated a similar reduction in Pol I transcription to HCT116 wild-type cells in <0.1% O_2_ (Supplementary Figure S2J). Therefore, the mechanism of repression of rDNA transcription is specific to hypoxia and is not mediated by the hypoxia-induced DDR. This led us to ask whether the repression of rDNA transcription was impacted by the concomitant accumulation of R-loops. To start testing this hypothesis, we investigated the oxygen dependency of R-loop accumulation in hypoxia and found that they do not accumulate in milder levels of hypoxia (2% O_2_), in contrast, exposure to <0.1% O_2_ led to R-loop accumulation to similar levels as seen in response to the DNA topoisomerase 1 poison camptothecin (CPT) (Figure 2H, I). Therefore the oxygen-dependency of R-loop accumulation and repression of rDNA transcription are similar. Together, these data demonstrate that radiobiological hypoxia (<0.1% O_2_) induces transcriptional stress which is evidenced by increased R-loops, decreased rDNA transcription and nucleolin translocation.

### Hypoxia-induced transcriptional stress is R-loop dependent

**Figure 3.**
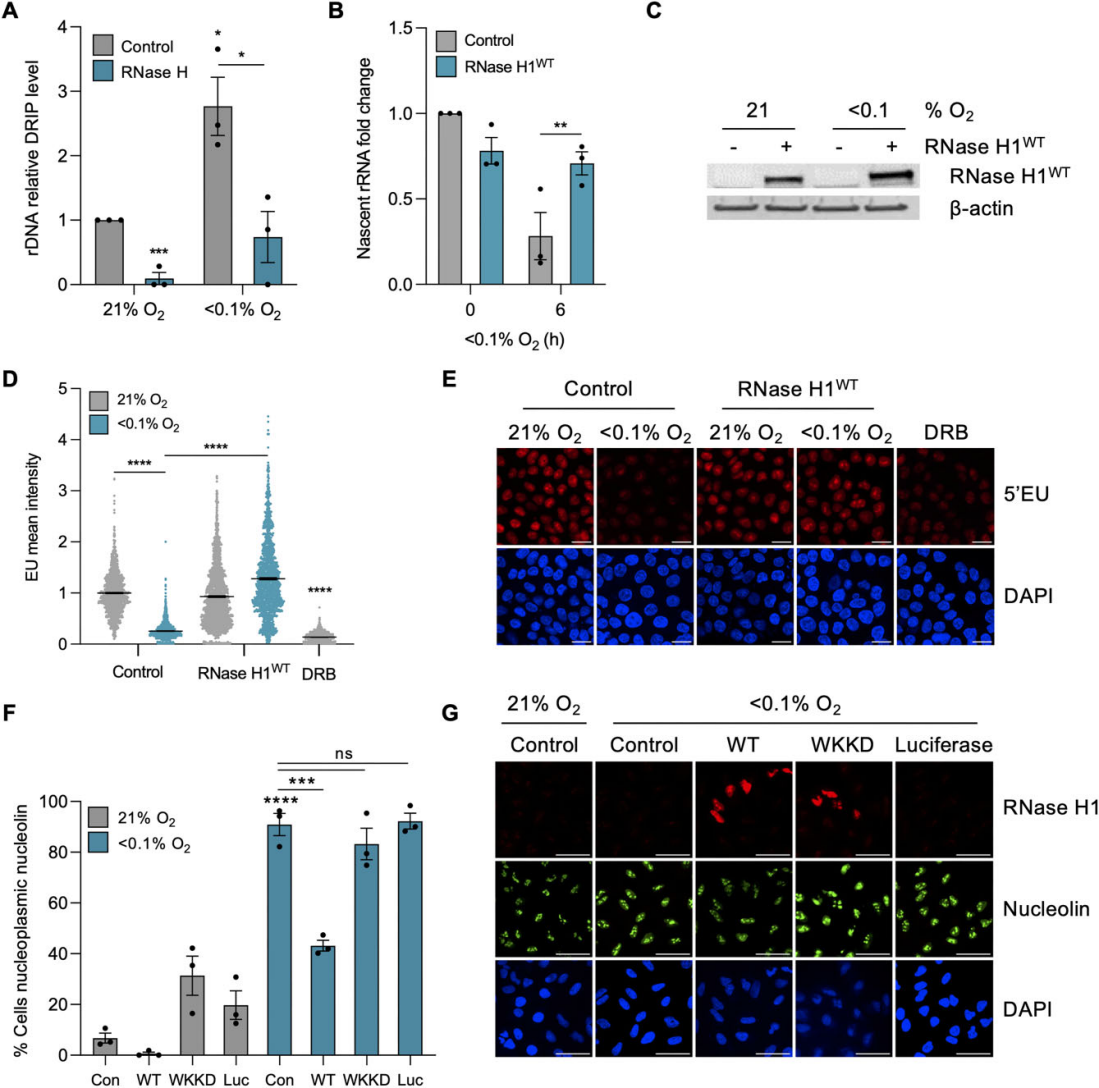
Hypoxia-induced transcriptional stress is R-loop dependent. (**A**) DRIP-qPCR analysis of A549 cells exposed to 21 or <0.1% O_2_ (6 h). Treatment with recombinant RNase H was used to confirm R-loop specificity. Values were normalized to the normoxic control sample. DRIP-qPCR ‘D1’ amplicon was used to analyze the promoter region of rDNA, as shown in S3A. (**B**) HCT116 cells were transfected with mock (control) or V5-tagged RNase H1^WT^ and exposed to 21 or <0.1% O_2_ (6 h). RT-qPCR for the nascent 47S rRNA precursor 47S is shown. Primers used to analyze the blue amplicon shown in Figure 2C were used. (**C**) HCT116 cells were transfected with mock (control) or V5-tagged RNase H1^WT^ and exposed to 21 or <0.1% O_2_ (6 h). RNase H1 over-expression was confirmed by western blot analysis. β-Actin was used as a loading control. (**D**) HCT116 cells were transfected with mock (control) or V5-tagged RNase H1^WT^, exposed to 21 or <0.1% O_2_ (6 h) and labeled with 5′EU (0.5 mM). The mean nuclear intensity of 5′EU per cell was quantified. DRB (100 μM, 1 h) was used as a control. The horizontal black bars represent the mean value, and each dot is one cell. (**E**) Representative images from part D showing EU (red) and DAPI (blue). Scale bar represents 20 μM. (**F**) A549 cells were transfected with mock (control), RNase H1^WT^, RNase H1^WKKD^ or Luciferase (Luc), exposed to 21 or <0.1% O_2_ (6 h), fixed and stained for nucleolin. The percentage of cells with nucleoplasmic nucleolin was quantified. (**G**) Representative images from part (F). V5 (red), nucleolin (green) and DAPI (blue) are shown. Scale bar represents 50 μM. (A–G) Data from three independent experiments (*n*= 3), mean ± standard error of the mean (SEM) are displayed unless otherwise indicated. * *P* < 0.05, ** *P* < 0.01, *** *P* < 0.001, **** *P* < 0.0001, ns (non-significant) *P* > 0.05. Unless otherwise indicated statistical significance refers to comparison to the normoxic control. In parts (A), (B) and (F), each data point represents the average from one of three biological repeats, normalized to the untreated sample. A minimum of 100 cells was imaged per condition in all microscopy experiments. The two-tailed, unpaired Student's *t*-test was used in parts (A), (B), (D) and (F).

Before further investigating mechanistic links between R-loops and hypoxia-induced transcriptional stress, we asked if R-loops accumulate at the rDNA in hypoxia. Using DNA/RNA immunoprecipitation (DRIP) followed by qPCR with primers to a region (D1), upstream of the main promoter on the rDNA (Supplementary Figure S3A), a significant increase in R-loops was detected in A549 and HCT116 cells (Figure 3A, Supplementary Figure S3B). Next, we asked how the markers of transcriptional stress we identified are impacted by reducing R-loops through RNase H1 over-expression in hypoxia. To determine how R-loop levels impacted Pol I-mediated transcription, we over-expressed RNase H1^WT^ and measured levels of the nascent 47S rRNA precursor. Transcription of the rDNA was significantly rescued by RNase H1^WT^ over-expression in hypoxia (Figure 3B, C and Supplementary Figure S3C). This was further supported by analyzing EU incorporation in the same conditions; again, over-expression of RNase H1^WT^ rescued global transcription (most of which can be attributed to rDNA transcription) in hypoxia (Figure 3D, E (HCT116) and Supplementary Figure S3D (A549)). Given that we already showed that the oxygen dependency of R-loop accumulation and nucleolin translocation did not match (Figure 2A, B), we predicted that reduction of R-loops would not impact nucleolin translocation in hypoxia. Surprisingly, we found that the proportion of cells with translocated nucleolin in hypoxia (<0.1% O_2_) was significantly reduced by over-expression of RNase H1^WT^. As controls, we over-expressed mutant RNase H1 (WKKD), which is unable to bind to R-loops or catalyze their resolution, and over-expressed an unrelated control plasmid (Luciferase) and found that neither rescued nucleolin translocation in hypoxia (Figure 3F, G) (44). Interestingly, this rescue was specific to radiobiological levels of hypoxia (<0.1% O_2_) as over-expression of RNase H1^WT^ did not rescue nucleolin translocation in mild levels of hypoxia (2% O_2_) (Supplementary Figure S3E, F). These data suggest that the mechanism of hypoxia-mediated translocation of nucleolin is oxygen-dependent; it is R-loop dependent in radiobiological hypoxia but R-loop independent in milder levels. Interestingly, nucleolin has been shown to bind both HIF-1α and R-loops (42,50,51). Our findings may reflect the two distinct stress responses; although HIF-1α is stabilized at both oxygen tensions, only radiobiological hypoxia leads to a DDR, UPR and R-loop accumulation (Supplementary Figure S3G). Together, these data demonstrate that the accumulation of R-loops in hypoxia is not only indicative of transcriptional stress but amplifies the stress response through reducing rDNA transcription and increasing nucleolin translocation.

### Hypoxia-induced R-loops are ROS dependent

**Figure 4.**
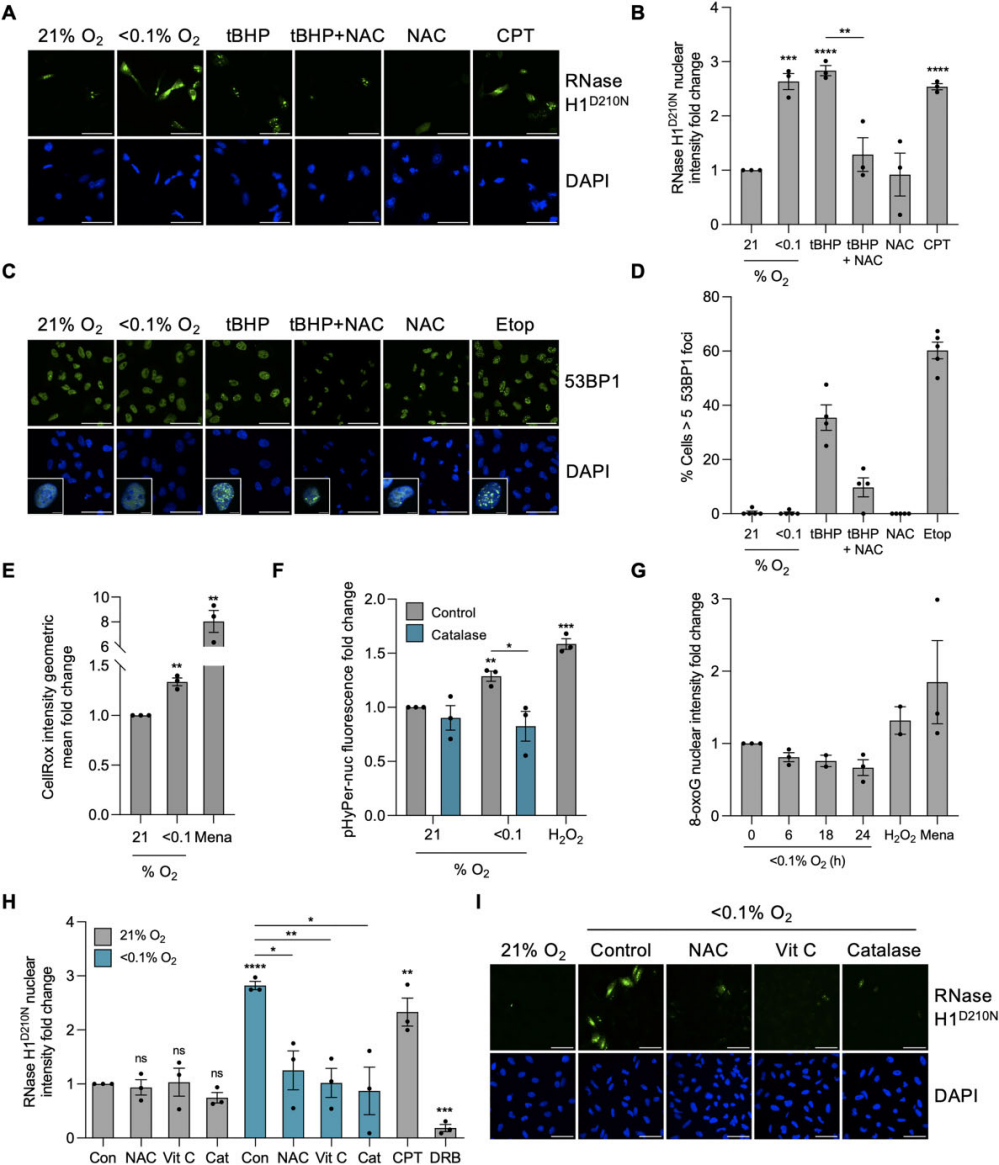
R-loop accumulation in hypoxia (<0.1% O_2_) is ROS-dependent. (**A**) A549 cells were transfected with V5-tagged RNase H1^D210N^ and exposed to 6 h of 21% O_2,_ <0.1% O_2,_ tBHP (20 μM), tBHP (20 μM) and NAC (20 mM), NAC (20 mM) or 1 h of CPT (10 μM). tBHP was used as a ROS-inducer, NAC was used as a ROS scavenger and CPT was used as a positive control for increasing R-loops. Cells were fixed and stained for V5 (green) and DAPI (blue). Scale bar represents 50 μM. (**B**) Quantification of V5-tagged RNase H1^D210N^ nuclear intensity from part A. (**C**) A549 cells were exposed to 6 h of 21% O_2,_ <0.1% O_2,_ tBHP (20 μM), tBHP (20 μM) and NAC (20 mM), or NAC (20 mM). Etoposide (Etop, 25 μM, 6 h) was used as a positive control for DNA damage. Cells were fixed and stained for 53BP1. Scale bar represents 50 μM. *n*= 1. (**D**) Quantification of part (C). Each data point represents a field of view of cells where percentage of cells with >5 53BP1 foci were determined. At least 100 cells were imaged per condition. *n*= 1. (**E**) A549 cells were exposed to 21, <0.1 or 2% O_2_ (6 h) with CellRox (5 μM) added during the last 10 min of treatment. Cells were fixed and CellRox intensity was measured. Menadione (100 μM, 6 h) was used as a positive control to increase ROS. At least 10 000 cells were quantified per condition. (**F**) A549 cells were transfected with pHyPer-Nuc and exposed to 21, <0.1 or 2% O_2_ (6 h) with and without catalase (2000 U/mg). H_2_O_2_ (5 mM, 3 h) was used as a positive control. Cells were fixed and the nuclear intensity was determined. (**G**) A549 cells were exposed to 21 or <0.1% O_2_ for the times indicated. Cells were fixed and stained for 8-oxoguanine (8-oxoG), and the nuclear intensity was determined. H_2_O_2_ (5 mM, 3 h) and menadione (100 μM, 3 h) were used as positive controls. (**H**) A549 cells were transfected with V5-tagged RNase H1^D210N^, treated with or without NAC (20 mM), Vitamin C (2 mM) or catalase (2000 U/mg) then exposed to 21 or <0.1% O_2_ (6 h). Cells were fixed and stained for V5. (**I**) Representative images from part H. V5 (green) and DAPI (blue) are shown. Scale bar represents 50 μM. (A–I) Data from three independent experiments (*n*= 3), mean ± standard error of the mean (SEM) are displayed unless otherwise indicated. * *P* < 0.05, ** *P* < 0.01, *** *P* < 0.001, **** *P* < 0.0001, ns (non-significant) *P* > 0.05. Unless otherwise indicated statistical significance refers to comparison to the normoxic control. In parts (B), (E)–(H), each data point represents the average from one of three biological repeats, normalized to the untreated sample. A minimum of 100 cells was imaged per condition in all microscopy experiments. The two-tailed, unpaired Student's *t*-test was used in parts (B), (E), (F) and (H).

We have shown previously that R-loops accumulate independently of DNA damage throughout the cell cycle and not specifically in hypoxic S-phase cells, leading us to conclude they do not arise as a result of TRCs (Supplementary Figure S4A, B) (10). Changes in redox have been linked to both the response to hypoxia and the accumulation of R-loops, which led us to question the role of ROS (52–66). To verify that increased ROS lead to R-loop accumulation, we treated cells with the oxidant, tert-Butyl hydroperoxide (tBHP), to increase ROS and then measured R-loop levels. R-loops accumulated in response to tBHP to similar levels as observed in hypoxic conditions and this increase in R-loops was rescued by use of the ROS scavenger *N*-acetyl-cysteine (NAC) (Figure 4A, B). However, ROS-induced R-loops are often associated with the concomitant induction of DNA damage (61,63,66). In agreement with these previous reports, we determined an increase in 53BP1 foci in response to tBHP. In contrast, hypoxia-induced R-loops occurred without an increase in 53BP1 foci (Figure 4C, D). Although increased ROS have been reported in hypoxia, few studies have measured ROS levels in conditions leading to transcriptional stress (<0.1% O_2_). Therefore, to confirm that hypoxia (<0.1% O_2_) does induce ROS, we used two complementary assays, one to measure total cellular ROS levels and the second to specifically measure nuclear H_2_O_2_. Firstly, we found total ROS levels increased in response to hypoxia (<0.1% O_2_) with menadione as a positive control (Figure 4E). Secondly, nuclear levels of H_2_O_2_ were found to increase and this increase was abrogated by the catalytic decomposition of H_2_O_2_ through the addition of catalase (Figure 4F). The presence of increased ROS in hypoxic conditions suggested the possibility of DNA damage in the form of oxidized bases, a form of DNA damage we have not previously investigated. However, we saw no accumulation of oxidized bases, measured by 8-oxoguanine (8-oxoG) in hypoxia suggesting that although ROS accumulate in hypoxia, they do not lead to detectable DNA damage (Figure 4G). Recently, non-DNA damaging levels of ROS were reported to lead to R-loop dependent replication stress, raising the hypothesis that hypoxia-induced ROS could still contribute to R-loop accumulation (67). We found that, treatment with three different ROS scavengers (NAC, vitamin C and catalase) completely abrogated R-loop accumulation in hypoxic conditions (Figure 4H, I). Together, these data suggest that increased ROS is the hypoxia-induced signal which leads to R-loop accumulation through a DNA damage-independent mechanism.

### Hypoxia-induced transcriptional stress is ROS dependent

**Figure 5.**
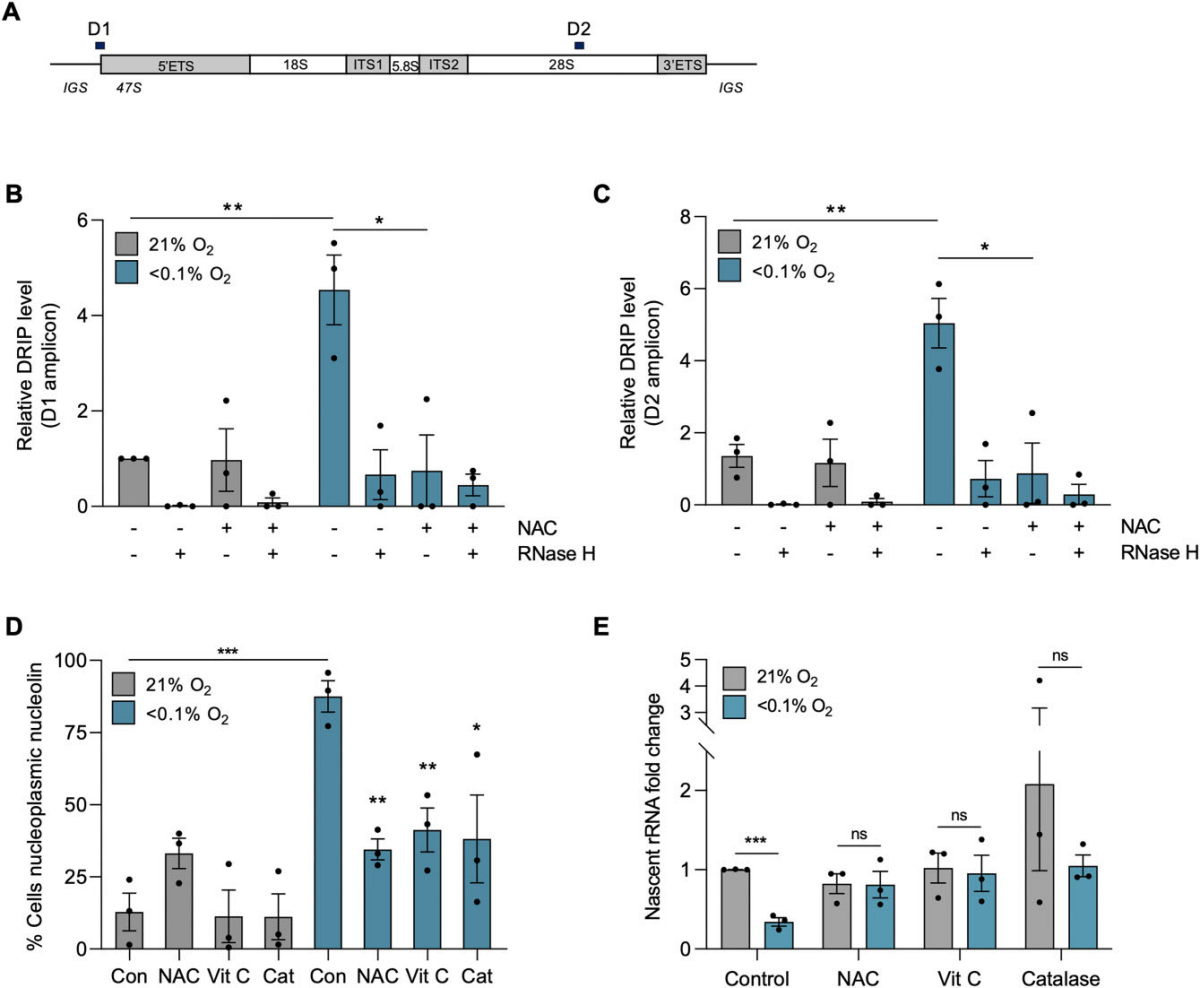
Hypoxia-induced transcriptional stress is ROS dependent. (**A**) rDNA repeat schematic with rDNA amplicons positions. D1 – 5′ rDNA promoter, D2 – 3′ 28S rRNA region. (**B**) DRIP-qPCR analysis of A549 cells exposed to 21 or <0.1% O_2_ (6 h), with and without NAC (20 mM). Treatment with recombinant RNase H was used to confirm R-loop specificity. The D1 amplicon was analyzed. Values were normalized to the normoxic control sample of D1. (**C**) as part B for D2 amplicon. Values were normalized to the normoxic control sample of D1. (**D**) A549 cells were treated with or without NAC (20 mM), Vitamin C (2 mM) or catalase (2000 U/mg), then exposed to 21 or <0.1% O_2_ (6 h), fixed and stained for nucleolin. The percentage of cells with nucleoplasmic nucleolin was quantified. Statistical significance is relative to the hypoxic (<0.1% O_2_) or normoxic (21% O_2_) control value. (**E**) A549 cells were treated with or without NAC (20 mM), Vitamin C (2 mM) or catalase (2000 U/mg) before being exposed to 21 or <0.1% O_2_ (6 h). RT-qPCR for the nascent 47S rRNA precursor is shown, normalized to untreated sample. (A–E) Data from three independent experiments (*n*= 3), mean ± standard error of the mean (SEM) are displayed unless otherwise indicated. * *P* < 0.05, ** *P* < 0.01, *** *P* < 0.001, **** *P* < 0.0001, ns (non-significant) *P* > 0.05. Unless otherwise indicated statistical significance refers to comparison to the normoxic control. In parts (B–E), each data point represents the average from one of three biological repeats. A minimum of 100 cells was imaged per condition in all microscopy experiments. The two-tailed, unpaired Student's *t*-test was used in parts (B) through (E).

To further support our conclusion that hypoxia-induced R-loops are ROS dependent, we carried out DRIP-qPCR analysis to determine the effect of reducing ROS on R-loop accumulation on the rDNA. Treatment with NAC abrogated the accumulation of R-loops on the rDNA in hypoxia (Figure 5A, B). To further support this finding we investigated an alternative region of the rDNA using primers that map to the 28S region (D2). Again, we observed that there was a robust accumulation of R-loops in hypoxia, and this accumulation was also significantly abrogated with NAC treatment (Figure 5C). Having further verified that R-loops accumulate in hypoxia in a ROS-dependent manner, we asked how the downstream markers of transcriptional stress were impacted by ROS levels. In the presence of ROS scavengers, the hypoxia-mediated translocation of nucleolin was significantly reduced (Figure 5D). In addition, repression of 47S transcription and EU incorporation in hypoxia were both rescued by ROS scavengers (Figure 5E (A549) and Supplementary Figure S4C (HCT116)). Together, these data demonstrate that hypoxia-induced transcriptional stress as determined by the accumulation of R-loops, nucleolin translocation and repression of rDNA transcription is ROS dependent.

### R-loops mediate rDNA transcription repression through heterochromatin formation

**Figure 6.**
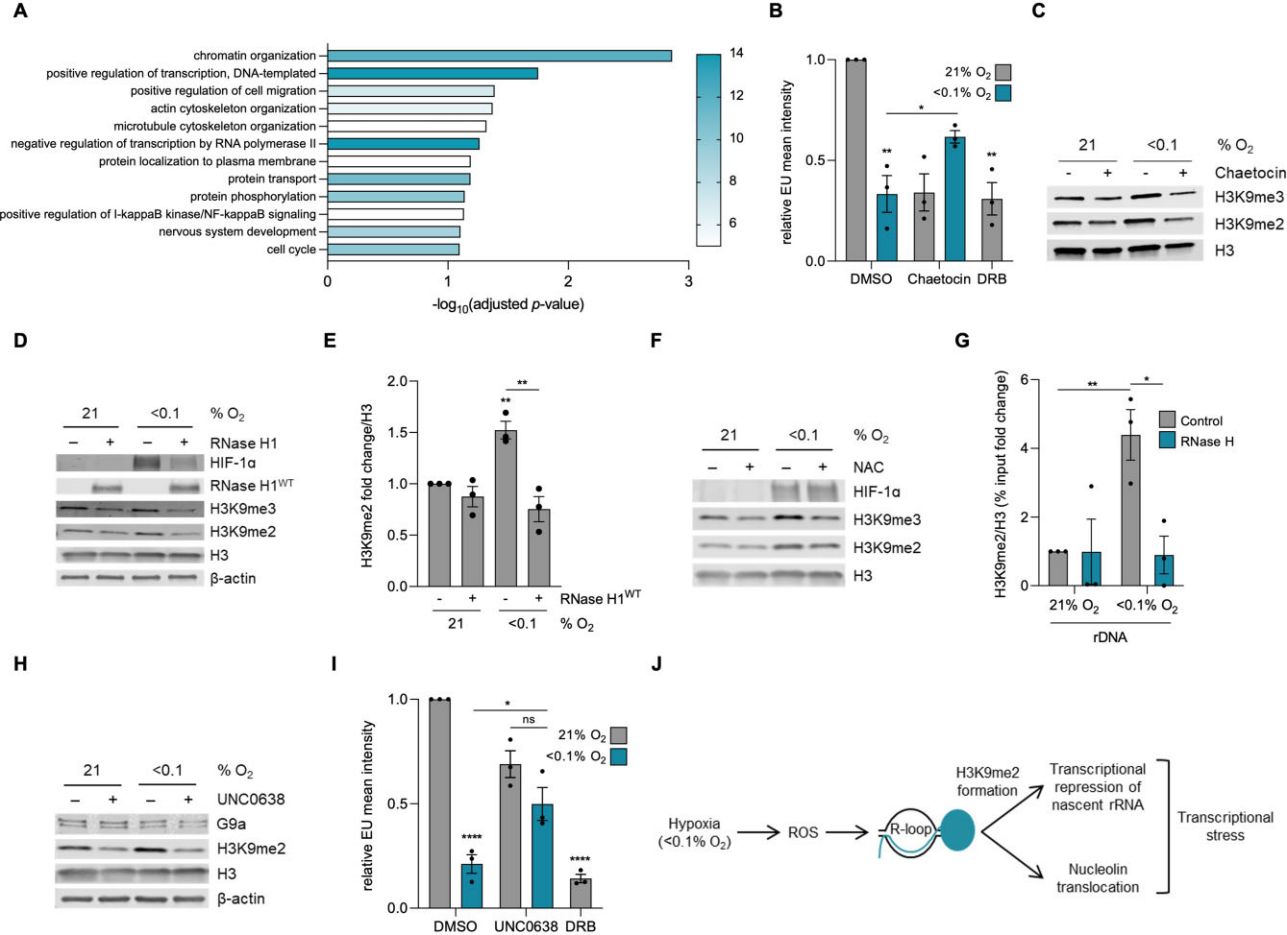
Hypoxia-induced R-loops repress rDNA transcription by mediating H3K9me2 formation. (**A**) Genes with the most similar expression pattern to SETX in TCGA LUAD and LUSC datasets were analyzed using GeneCodis. The top 12 most significantly enriched pathways, from gene ontology biological pathway analysis, are shown. Color represents number of genes in the pathway. Genes in the ‘chromatin organization’ pathway are shown in Supplementary Table S2. (**B**) HCT116 cells were treated with DMSO or Chaetocin (1 μM), exposed to 21 or <0.1% O_2_ (6 h), and labeled with 5′EU (0.5 mM). The mean nuclear intensity per cell of 5′EU was quantified. DRB (100 μM, 6 h) was used as a control. Each data point represents the average 5′EU intensity from each of the three biological repeats. The two-tailed, unpaired Student's *t*-test was used. (**C**) HCT116 cells were treated with and without Chaetocin (1 μM) and exposed to 21 or <0.1% O_2_ (6 h), followed by western blotting. H3 was used as a loading control. Quantification of the western blot is shown in Supplementary Figure S5A, B. (**D**) A549 cells were transfected with mock or V5-tagged RNase H1^WT^ and exposed to 21 or <0.1% O_2_ (4 h). RNase H1 over-expression was confirmed by western blot analysis. (**E**) Quantification of H3K9me2 from part D. The two-tailed, unpaired Student's *t*-test was used. Quantification of H3K9me3 is shown in Supplementary Figure S5C. (**F**) A549 cells were treated with NAC (20 mM) and exposed to 21 or <0.1% O_2_ (6 h), followed by western blotting. H3 was used as a loading control. Quantification of the western blot is shown in Supplementary Figure S5D, E. (**G**) ChIP-qPCR analysis of H3K9me2/H3 levels in A549 cells over-expressing RNase H1^WT^ exposed to 21 or <0.1% O_2_ (6 h). The rDNA (D1) amplicon was analyzed. Values are calculated as a percentage of input, subtracted from the no antibody control value, and normalized to the D1 normoxic (21% O_2_) value. (**H**) A549 cells were pre-treated with UNC0638 (1 μM, 72 h) then exposed to 21 or <0.1% O_2_ (4 h) followed by western blotting. β-Actin and H3 were used as a loading control. Both the 165 kDa G9a-L and 140 kDa G9a-S isoforms are shown. (**I**) A549 cells were pre-treated with UNC0638 (3 μM, 20 h) then exposed to 21 or <0.1% O_2_ (6 h) and labeled with 5′EU (0.5 mM). The mean nuclear intensity of 5′EU was quantified. DRB (100 μM, 6 h) was used as a control. Each data point represents the average 5′EU intensity from each of the 3 biological repeats. The two-tailed, unpaired Student's *t*-test was used. (**J**) Hypoxia (<0.1% O_2_) leads to an increase in ROS which causes an accumulation of R-loops that contribute to the transcriptional stress response. The transcriptional stress response includes R-loop dependent translocation of nucleolin, and R-loop dependent deposition of H3K9me2 on rDNA that represses rDNA transcription. (A–J) Data from three independent experiments (*n*= 3), mean ± standard error of the mean (SEM) are displayed unless otherwise indicated. * *P* < 0.05, ** *P* < 0.01, *** *P* < 0.001, **** *P* < 0.0001, ns (non-significant) *P* > 0.05. Unless otherwise indicated statistical significance refers to comparison to the normoxic control. In parts (B), (E), (G) and (I), each data point represents the average from one of three biological repeats, normalized to the untreated sample. A minimum of 100 cells was imaged per condition in all microscopy experiments. The two-tailed, unpaired Student's *t*-test was used in parts (B), (E), (G) and (I).

Next, we sought to investigate the mechanism underpinning R-loop mediated repression of rDNA transcription. We demonstrated previously that the expression of the hypoxia-induced RNA/DNA helicase SETX correlates with expression of genes which have also been shown to be induced in response to radiobiological hypoxia (<0.1% O_2_) (10). Here, as a means of identifying potential mechanisms to link R-loops to repression of Pol I activity, we used patient samples from lung squamous cell carcinoma and lung adenocarcinoma (LUAD, LUSC) in the Cancer Genome Atlas Program (TCGA) datasets to ask which genes were expressed in the most similar pattern to SETX and which biological pathways those genes impacted (37,38,68). We found that the most significant (*P*-value = 0.001373) pathway association was with chromatin organization (Figure 6A) (39). Of the 12 genes enriched in this pathway, 3 are involved in general histone methylation, and 2 are specifically involved in the methylation of H3K9 (Supplementary Table S2). R-loops have been shown to lead to changes in chromatin marks, including H3K9me2, and in some cases this has been linked to transcriptional repression (10,68–70). We asked if changes at the chromatin level could provide the mechanism of R-loop mediated repression of rDNA transcription and began by using Chaetocin, which inhibits both the Suv39h1 and G9a methyltransferases (71,72). In response to Chaetocin treatment, EU incorporation increased in hypoxic conditions supporting a link between chromatin and transcription (Figure 6B, C, quantified changes in H3K9me2 and me3 are shown in Supplementary Figure S5A, B). Both H3K9me2 and me3 have been shown to increase in response to hypoxia and importantly, we demonstrated previously that over-expression of RNase H1^WT^ decreased H3K9me2 and me3 accumulation in hypoxia in HCT116 cells (10,73). Here, we verified that in a second cell line, A549, hypoxia-induced H3K9me2 was significantly reduced by over-expression of RNase H1^WT^ (Figure 6D, E). A noticeable reduction in hypoxia-induced H3K9me3 was observed, however this was not statistically significant in A549 cells (*P* = 0.0794) (quantification shown in Supplementary Figure S5C). Together these data support a model where hypoxia-induced R-loops lead to the accumulation of H3K9me2 and likely H3K9me3 and that this impacts transcription rates. We have shown that R-loops accumulate in hypoxia in a ROS-dependent manner and therefore asked if scavenging ROS would reduce H3K9me2/3 in hypoxia. The addition of NAC significantly reduced the accumulation of both H3K9me2 and H3K9me3 in hypoxia (Figure 6F and quantification shown in Supplementary Figure S5D, E). To further support our model that hypoxia-induced R-loops lead to increases in H3K9me2/3 and that this is linked to repression of rDNA synthesis, we asked if H3K9me2 accumulated at the rDNA in hypoxia. We chose to focus on H3K9me2 as both our data and previous reports are more supportive of this mark being involved (69,74). Using ChIP, we found H3K9me2 accumulates on the rDNA in the same region we previously saw R-loops increase, and this was abrogated by RNase H1^WT^ over-expression (Figure 6G). Next, we investigated the G9a methyltransferase using a specific inhibitor (UNC0638) and found that hypoxia-induced H3K9me2 was G9a dependent (Figure 6H). As controls, we verified that the use of Chaetocin or UNC0638 did not impact R-loop accumulation in hypoxia (Supplementary Figure S5F). This is in line with previous studies which reported that a decrease in H3K9me2 did not impact R-loop levels (69,70). Having implicated G9a as the methyltransferase responsible for H3K9me2 accumulation in hypoxia, we asked if inhibition of G9a would also impact transcription in hypoxia. Using UNC0638 to inhibit G9a, we found that transcription was significantly rescued in hypoxia (Figure 6I). Overall, these data support a model that hypoxia-induced ROS lead to R-loops that accumulate on the rDNA, increase the heterochromatic H3K9me2 mark, leading to reduced rRNA transcription (Figure 6J).

## Discussion

This study characterizes hypoxia-induced transcriptional stress and demonstrates that ROS-induced R-loops play a critical role in this response. We found that the consequence of R-loop accumulation in hypoxia includes the translocation of nucleolar stress response protein nucleolin, as well as the deposition of the heterochromatic mark, H3K9me2 at the rDNA leading to repression of Pol I-mediated transcription. Together, these data suggest a role for hypoxia-induced R-loops in downregulating the most energy-consuming cellular process, ribosome biogenesis, by repressing Pol I-mediated production of rRNA. The link between ROS and increased R-loops in hypoxia is consistent with an energy conservation role as ROS accumulate throughout the cell cycle. Increases in ROS in hypoxia have been described and are primarily attributed to electron leakage from the mitochondrial electron transport chain. Moreover, one of the roles of UPR is to restrict ROS levels through PERK/NRF2 signaling, possibly explaining the link between UPR and R-loops in hypoxia which demonstrates that in the absence of the UPR, R-loops accumulate further.

Nucleolin has previously been described as a multifaceted protein and responds to a myriad of stresses including heat shock, replication stress and DNA damage (75,76). Our data demonstrates that nucleolin translocation occurs in a range of hypoxic conditions (<0.1–2% O_2_) and that this is only R-loop dependent at the lower/radiobiological level (<0.1% O_2_). These findings suggest that nucleolin translocation is a generic response to adverse conditions and that the underlying mechanisms are stress-specific. It is interesting to consider how HIF-1α may play a role as nucleolin has been shown to bind to and stabilize HIF-1α, however this raises questions about how the HIF response differs in mild versus radiobiological hypoxia (50).

We have identified a ROS/R-loops/transcriptional stress response axis in hypoxia, however the mechanism by which ROS induce R-loops remains unclear. Previous reports have shown that ROS lead to DNA damage in the form of SSBs and oxidized bases, which physically impede the transcription machinery and lead to an accumulation of R-loops (61,63,66). However, we have not been able to detect markers of DNA damage, including oxidized bases, in hypoxic conditions. In support of our findings, low levels of H_2_O_2,_ which did not lead to detectable DNA damage, have been shown to cause Pol II stalling and polymerase stalling has been described as both a cause and consequence of R-loop formation (43,44,77,78). This suggests the hypothesis that hypoxia-induced ROS may cause Pol I to stall and form R-loops, which recruit methyltransferases, including G9a, to deposit the heterochromatic H3K9me2, blocking new Pol I from entering promoters and transcribing rDNA. A recent study demonstrated that ROS generated in response to hydroxyurea-induced replication fork slowdown lead to R-loops without causing DNA damage, consistent with our study that suggests ROS can lead to an accumulation of R-loops without causing SSBs/DSBs (67). However, this study also showed that the eventual replication fork collapse, as an indirect consequence of R-loop accumulation, led to DNA damage. Furthermore, consideration should be given to the hypothesis that hypoxia-induced ROS, while not damaging the DNA, could be damaging or inhibitory to critical stress-response proteins through direct oxidation. Moreover, it would be interesting to see whether hypoxic cells treated with G9a inhibitor or over-expressing RNase H1^WT^ and therefore maintaining transcription of the rDNA accumulate DNA damage as a result of more TRCs.

Another study showed that in *BRCA2* deficient cells, elevated levels of ROS impair RNase H1, which leads to an accumulation of R-loops on the mitochondrial DNA (64). We have recently shown that chromatin accessibility decreases in hypoxia, including decreased accessibility at promoters for factors identified in the R-loop interactome (12). Together this suggested that hypoxia-induced ROS could impact the expression of factors with key roles in R-loop resolution leading to their persistence. However, it should be noted that restoring specific R-loop factors in hypoxia did not universally reduce R-loop accumulation for example, rescuing DDX5 further increased R-loops in hypoxia while rescuing DHX9 reduced R-loop levels, implying that the accumulation of R-loops in hypoxia may not simply be attributed to the repression of the R-loop interactome (12).

Changes in liquid–liquid phase separation (LLPS) in hypoxia offer another possible hypothesis for how ROS leads to R-loops. Sources of oxidative stress have been shown to lead to LLPS in the cell, allowing for structures such as stress granules and glycolytic bodies to form (79). Moreover, multiple R-loop binding proteins have been shown to contain intrinsically disordered regions that allow them to undergo LLPS under certain cellular conditions (80). Interestingly, phase separation has been shown to repress rDNA transcription (81). Therefore, one hypothesis is that in hypoxia, R-loop resolving enzymes undergo LLPS and no longer resolve R-loops, leading them to accumulate.

Studies have linked R-loops to the formation of heterochromatin marks, and one study showed that nutrient deprivation leads to H3K9me2 which contributes to rDNA silencing (43,69,70,82–84). Supportively, nutrient deprivation has also been shown to increase total cellular ROS levels, therefore it would be interesting to investigate whether general cellular stresses such as nutrient deprivation, heat shock, UV also repress rDNA transcription through ROS-induced R-loops (85).

Hyper-transcription of rDNA, elevated ribosome biogenesis and dysregulated expression of nucleolar proteins such as nucleolin are features of many cancers (86). It is plausible that hypoxia exerts a selection pressure to select for cells that can maintain relatively high levels of rDNA transcription. Hypoxic tumors are associated with loss of the tumor suppressor *PTEN* and gain of oncogene *MYC*, both of which have been linked to hyperactivated production of rRNA and ribosomes (87–92). Moreover, a correlation between alterations in the KEAP-NRF2 master antioxidant system and higher hypoxia scores in patient samples has been observed, demonstrating that hypoxic tumors select for cells that can counteract ROS through constitutive expression of NRF2 (89,93). Furthermore, a likely consequence of hypoxia is that upon reoxygenation, DNA damage can accumulate in the rDNA as a result of restored transcription, allowing for more TRCs, and as a result of a significant increase in ROS to DNA-damaging levels. Therefore, disrupted rDNA transcription in hypoxia, followed by sudden reoxygenation, can contribute to genomic instability in the tumor. Overall, our study has provided a novel mechanistic insight into understanding how hypoxic cells regulate transcriptional stress through an ROS–R-loop–H3K9me2 axis and offer a model for understanding how cancers might exploit and dysregulate this axis to evade repression of ribosome biogenesis and to maintain high proliferation rates.

## Supplementary Material

gkad858_Supplemental_File

## Data Availability

All data is available from the corresponding author upon request.
