# Supplementary material for: Hypoxia-induced transcriptional stress is mediated by ROS-induced R-loops
Source: Nucleic Acids Res. 2023 Oct 16;51(21):11584–99. doi: 10.1093/nar/gkad858 (PMC10681727; doi:10.1093/nar/gkad858)
Supplement: gkad858_Supplemental_File [file gkad858_supplemental_file.pdf]

Hypoxia-induced transcriptional stress is mediated by ROS-induced R-loops

Tiffany S. Ma[1], Katja R. Worth[1], Conor Maher[1], Natalie Ng[1], Chiara Beghè[2], Natalia Gromak[2], Anna M. Rose[3], and Ester M. Hammond[1]*

[1]Department of Oncology, University of Oxford, Oxford, OX3 7DQ, UK. [2]Sir William Dunn School of Pathology, University of Oxford, OX1 3RE, Oxford, UK [3]Department of Pediatrics, University of Oxford, Oxford OX3 9DU, UK

*Corresponding author

Ester.Hammond@oncology.ox.ac.uk

SI Contents

Table S1 – Primer/probe sequences used	Page 2
Table S2 – Other GO Biological Processes of the 12 genes in ‘chromatin organization’ pathway	Page 2
SI Methods	Page 6
SI Figure legends	Page 6

Table S1 – Primer/probe sequences used

Primer/probe	Sequence	Application
18S	**F:** GCCCGAAGCGTTTACTTTGA<br>**R:** TCCATTATTCCTAGCTGCGGTATC	RT-qPCR
Nascent 47S rRNA precursor	**F:** ACCTGTCGTCGGAGAGGTTG<br>**R:** GTCACCGTGAGGCCAGAG	RT-qPCR
XBP1-sp	**F:** TGCTGAGTCCGCAGCAGGTG<br>**R:** GCTGGCAGGCTCTGGGGAAG	RT-qPCR
Nascent rRNA (N1)	CGGAGGCCCAACCTCTCCGACGACAGGTCGCCAGAGGA CAGCGTGTCAGC	Northern
rDNA (D1 amplicon)	**F:** GGTATATCTTTCGCTCCGAGTC<br>**R:** ACAGGTCGCCAGAGGACAG	DRIP; ChIP
rDNA (D2 amplicon)	**F:** TGGCGCTAAACCATTCGTAG<br>**R:** GTCGAGGGCTGACTTTCAATAG	DRIP

Table S2 – Other GO Biological Processes of the 12 genes in 'chromatin organization' pathway

Gene name	GO Biological Processes
ASH1L	histone H3-K9 methylation<br>histone H3-K4 methylation<br>histone H3-K36 dimethylation<br>methylation<br>post-embryonic development<br>uterine gland development<br>tarsal gland development<br>skeletal system development<br>regulation of gene expression<br>uterus morphogenesis<br>negative regulation of acute inflammatory response<br>negative regulation of I-kappaB kinase/NF-kappaB signaling<br>sebaceous gland development<br>flagellated sperm motility<br>positive regulation of transcription by RNA polymerase II<br>decidualization<br>negative regulation of MAPK cascade<br>single fertilization<br>negative regulation of inflammatory response
BRD3	regulation of transcription by RNA polymerase II<br>regulation of transcription, DNA-templated<br>chromatin remodeling
CHD1	inactivation of X chromosome by heterochromatin assembly<br>negative regulation of double-strand break repair via homologous

	recombination<br>positive regulation of double-strand break repair via nonhomologous end joining<br>nose development<br>positive regulation of DNA repair<br>chromosome organization<br>positive regulation by host of viral transcription<br>DNA duplex unwinding<br>dosage compensation by inactivation of X chromosome<br>chromatin remodeling<br>cellular response to DNA damage stimulus<br>DNA repair<br>double-strand break repair
EHMT1	histone H3-K9 methylation<br>histone methylation<br>histone lysine methylation<br>histone H3-K27 methylation<br>peptidyl-lysine monomethylation<br>peptidyl-lysine dimethylation<br>DNA methylation<br>methylation<br>regulation of embryonic development<br>negative regulation of transcription by RNA polymerase II<br>negative regulation of transcription, DNA-templated<br>positive regulation of cold-induced thermogenesis
KDM3B	histone H3-K9 demethylation<br>regulation of transcription by RNA polymerase II
NCOR1	negative regulation of transcription by RNA polymerase II<br>negative regulation of JNK cascade<br>regulation of transcription by RNA polymerase II<br>negative regulation of fatty acid metabolic process<br>spindle assembly<br>negative regulation of transcription, DNA-templated<br>negative regulation of androgen receptor signaling pathway<br>negative regulation of glycolytic process<br>locomotor rhythm<br>negative regulation of production of miRNAs involved in gene silencing by miRNA<br>rhythmic process
NSD1	histone methylation<br>histone lysine methylation<br>histone H4-K20 methylation<br>histone lysine methylation<br>methylation<br>regulation of RNA polymerase II regulatory region sequence-specific DNA

	binding<br>regulation of histone H3-K36 methylation<br>regulation of transcription, DNA-templated<br>positive regulation of transcription, DNA-templated<br>negative regulation of transcription by RNA polymerase II
PHF2	histone H4-K20 demethylation<br>protein demethylation<br>histone H3-K9 demethylation<br>positive regulation of transcription, DNA-templated<br>negative regulation of ribosomal DNA heterochromatin assembly<br>liver development<br>epigenetic maintenance of chromatin in transcription-competent conformation<br>regulation of transcription by RNA polymerase II
RNF20	positive regulation of histone methylation<br>positive regulation of transcription, DNA-templated<br>protein ubiquitination<br>histone H2B conserved C-terminal lysine ubiquitination<br>positive regulation of histone H2B ubiquitination<br>ubiquitin-dependent protein catabolic process<br>regulation of mitotic cell cycle<br>regulation of transcription, DNA-templated<br>protein polyubiquitination<br>negative regulation of mRNA polyadenylation<br>histone H2B ubiquitination<br>histone monoubiquitination<br>histone ubiquitination<br>negative regulation of cell migration
SMARCA2	positive regulation of transcription, DNA-templated<br>negative regulation of transcription by RNA polymerase II<br>nervous system development<br>regulation of transcription by RNA polymerase II<br>spermatid development<br>negative regulation of transcription, DNA-templated<br>negative regulation of cell growth<br>negative regulation of cell population proliferation<br>positive regulation of transcription by RNA polymerase II<br>chromatin remodeling<br>regulation of transcription, DNA-templated
SMCHD1	inactivation of X chromosome by heterochromatin assembly<br>negative regulation of double-strand break repair via homologous recombination<br>positive regulation of double-strand break repair via nonhomologous end joining<br>nose development

	double-strand break repair<br>positive regulation of DNA repair<br>chromosome organization<br>dosage compensation by inactivation of X chromosome<br>cellular response to DNA damage stimulus<br>DNA repair
ZNF462	regulation of gene expression<br>positive regulation of transcription by RNA polymerase II<br>negative regulation of DNA binding

SI METHODS

Northern blotting

2-3 µg of glyoxal-treated total RNA was resolved on a 1% sodium phosphate gel. Agarose gels were transferred onto positively charged nylon membranes by capillarity overnight in 20x saline sodium citrate (SCC). UV- crosslinked membranes were prehybridized in Church Buffer at 65°C for 1 hour. The ^{32}P-labeled oligonucleotide probe was incubated with the membrane overnight at 65°C. Probe sequence for 'N1' can be found in **Table S1**.

SI FIGURE LEGENDS

Figure S1. Hypoxia leads to accumulation of nucleolar R-loops

A. A549 cells were treated with Thaps (2 µM) or Tuni (5 µg/mL) for the times indicated followed by western blotting for UPR markers, PERK and GRP78. An electrophoretic shift in PERK indicates an activated UPR. β-actin was used as a loading control.

B. A549 cells were transfected with V5-tagged RNase H1^{D210N} and treated with Thaps (2 µM, 6 h) or Tuni (5 µg/mL, 6 h). Cells were fixed and stained for V5 and the nuclear intensity was determined. CPT (10 µM, 1 h) and DRB (100 µM, 1 h) were used as controls to increase and decrease R-loop levels, respectively. Each data point represents the average from one of multiple biological repeats, normalized to the untreated sample. *n*=3, except for control and Thaps *n*=4

C. A549 cells were treated with IRE1α inhibitor (4µ8c, 20 µM) and exposed to <0.1% O_2 for the times indicated, followed by RT-qPCR for the spliced XBP1. The data point shown represents the average from three technical replicates from one biological repeat. *n*=1

D. A549 cells were treated with PERK inhibitor (AMG PERK 44, 20 µM), exposed to <0.1% O_2 for the times indicated followed by western blotting for PERK. β-actin was used as a loading control.

E. A549 cells were transfected with V5-tagged RNase H1^{D210N} and exposed to 21 or <0.1% O_2 (6 h). Cells were fixed and co-stained for V5 (green), the nucleolar marker fibrillarin (red) and DAPI (blue). CPT

(10 µM, 1 h) and DRB (100 µM, 1 h) were used as controls for increasing and decreasing R-loops, respectively. Scale bar represents 50 µM. *n*=1 An enlarged cell is shown in Figure 1C.

F. HCT116 cells were exposed <0.1% O_2 (1.5 h). Cells were fixed and co-stained for S9.6 (green), nucleolar marker nucleolin (red) and DAPI (blue). Coverslips were treated with recombinant RNase H as a control to abolish S9.6 signal. Scale bar represents 2.5 µM. Quantification is shown in Figure 1D.

G. RKO cells were exposed to 21, 2 or <0.1% O_2 (6 h) and labeled with 5'EU (0.5 mM). The mean nuclear intensity of 5'EU per cell was quantified. DRB (100 µM, 6 h) was used as a control.

H. MRC5 cells were exposed to 21, 2 or <0.1% O_2 (6 h) and labeled with 5'EU (0.5 mM). The mean nuclear intensity of 5'EU per cell was quantified. DRB (100 µM, 6 h) was used as a control.

I. $HCT116^{HIF\text{-}1\alpha+/+}$ and $HCT116^{HIF\text{-}1\alpha-/-}$ cells from Figure 1G were exposed to <0.1% O_2 for the times indicated. HIF-1α knockout was confirmed by western blot analysis. H3 was used as a loading control.

A-I Data from three independent experiments (*n*=3), mean ± standard error of the mean (SEM) are displayed unless otherwise indicated. * p <0.05, ** p <0.01, *** p <0.001, **** p <0.0001, ns (non-significant) p >0.05. Unless otherwise indicated statistical significance refers to comparison to the normoxic control. In parts **G** and **H**, each dot represents a cell. A minimum of 100 cells was imaged per condition in all microscopy experiments. The two-tailed, unpaired Student's *t*-test was used in parts **B, G** and **H.**

Figure S2. Hypoxia-mediated repression of 47S RNA synthesis is independent of the DDR

A. A549 cells were exposed to <0.1% O_2 for the times indicated followed by western blotting. β-actin was used as a loading control.

B. A549 cells were exposed to 21, 2 or <0.1% O_2 (18 h) followed by RT-qPCR for the nascent 47S rRNA precursor. The rDNA transcription inhibitors Actinomycin D (Act D, 40 nM, 6 h), CX5461 (CX, 100 nM, 6 h), and BMH-21 (BMH, 1 mM, 6 h) were used as controls. The data point shown represents the average from three technical replicates from one biological repeat. *n*=1

C. Schematic representing transcription unit of the human rDNA locus. Transcription of the rDNA by Pol I results in a production of a long 47S rRNA precursor. Cleavage at the initial site A' on the 5'ETS (5'externally transcribed spacer) region and 02 on the 3'ETS (3'externally transcribed spacer) region results in the production of a shorter transcript 45S which is further processed to form the mature 18S, 5.8S and 28S. ITS1, internally transcribed spacer 1; ITS2, internally transcribed spacer 2. The location of primers/probes used in the subsequent experiments is indicated.

D. HCT116 cells were exposed to <0.1% O_2 for the times indicated or reoxygenated (<0.1% O_2 followed by 21% O_2, 1 h). Northern blot using northern probe N1 (brown box, Figure S2C) of the 5'ETS region is shown. CX5461 (CX, 100 nM, 6 h) was used as a control. MB = methylene blue (loading control). *n*=1

E. HCT116 cells were pre-treated with ATM inhibitor AZD1390 (1 h) at the concentrations indicated before being exposed to <0.1% O_2 (2.5 h). Western blotting was carried out as indicated. β-actin was used as a loading control.

F. HCT116 cells were pre-treated with ATM inhibitor KU55933 (10 µM, 1 h) before being exposed to 21 or <0.1% O_2 (3 h). Inhibition of ATM signaling was confirmed by western blot analysis of KAP1-S824. β-actin was used as a loading control.

G. HCT116 cells were treated with ATM inhibitor KU55933 (10 µM, 1 h) before being exposed to <0.1% O_2 for the times indicated. RT-qPCR for the nascent 47S rRNA precursor is shown. Primers denoted by the blue box in S2C, located in the upstream promoter region of 47S, were used.

H. HCT116 cells were pre-treated with ATR inhibitor AZD6738 (1 nM, 1 h) before being exposed to 21 or <0.1% O_2 (6 h). Inhibition of ATR signaling was confirmed by western blot analysis of Chk1-S345. β-actin was used as a loading control.

I. HCT116$^{ATR\ WT}$ and HCT116$^{ATR/\text{-flox}}$ were exposed to 21 or <0.1% O_2 (4 h). ATR protein levels were confirmed by western blot analysis. β-actin was used as a loading control.

J. HCT116$^{ATR\ WT}$ and HCT116$^{ATR/\text{-flox}}$ cells were exposed to 21 or <0.1% O_2 for the times indicated. RT-qPCR for the nascent 47S rRNA precursor, normalized to the normoxic HCT116$^{ATR\ WT}$ control is shown.

Primers used to analyze the blue amplicon in part C, located in the upstream promoter region of 47S, were used. *n*=2

A-J Data from three independent experiments (*n*=3), mean ± standard error of the mean (SEM) are displayed unless otherwise indicated. * p <0.05, ** p <0.01, *** p <0.001, **** p <0.0001, ns (non-significant) p >0.05. Unless otherwise indicated statistical significance refers to comparison to the normoxic control. In parts **G** and **J**, each data point represents the average from one of multiple biological repeats, normalized to the untreated sample. The two-tailed, unpaired Student's *t*-test was used in parts **G** and **J**.

Figure S3. Hypoxia-induced transcriptional stress is oxygen-dependent

A. Schematic representation of the rDNA transcriptional unit in humans. The location of D1 amplicon on the nascent 47S rRNA precursor, is indicated. IGS denotes intergenic spacer. Each data point represents the average from one of three biological repeats, normalized to the untreated sample.

B. DRIP-qPCR analysis of HCT116 cells exposed to 21 or <0.1% O_2 (1.5 h). CPT (10 µM, 1 h) was used as a control to increase R-loops. Primers analyzing the D1 amplicon in part A were used. Treatment with recombinant RNase H was used to confirm R-loop specificity. Values were normalized to the normoxic control sample. The two-tailed, unpaired Student's *t*-test was used.

C. A549 cells were transfected with mock (control) or RNase H1^{WT} and exposed to 21 or <0.1% O_2 (6 h). RT-qPCR for the nascent 47S rRNA precursor is shown, normalized to the normoxic control sample. *n*=1

D. A549 cells were transfected with mock (control) or RNase H1^{WT}, exposed to 21 or <0.1% O_2 (6 h) and labeled with 5'EU (0.5 mM). The mean nuclear intensity of 5'EU per cell was quantified. DRB (100 µM, 1 h) was used as a control. Each dot represents a cell, and the horizontal black line denotes the average of mean nuclear intensities. *n*=1

E. A549 cells were transfected with mock (control) or V5-tagged RNase H1^{WT}, exposed to 21 or 2% O_2 (6 h), fixed and stained for nucleolin, V5 and DAPI. The percentage of transfected cells with nucleoplasmic nucleolin was quantified. The black dot represents the average of multiple fields of views from one biological repeat. *n*=1

F. Representative images from part E. Nucleolin (green), V5 (red) and DAPI (blue) are shown. Scale bar represents 50 μM.

G. A549 cells were exposed to <0.1 or 2% O_2 for the times indicated. Western blotting was carried out for HIF-1α, UPR markers (PERK, GRP78) and DDR markers (p53, p53-S15, H2AX, γ-H2AX). β-actin was used as a loading control.

A-G Data from three independent experiments (*n*=3), mean ± standard error of the mean (SEM) are displayed unless otherwise indicated. * $p < 0.05$, ** $p < 0.01$, *** $p < 0.001$, **** $p < 0.0001$, ns (non-significant) $p > 0.05$. Unless otherwise indicated statistical significance refers to comparison to the normoxic control. In parts **C** and **E**, each data point represents the average from one biological repeat. A minimum of 100 cells was imaged per condition in all microscopy experiments.

Figure S4. Hypoxia-mediated repression of rDNA transcription is ROS dependent in the absence of DNA damage

A. HCT116 cells were exposed to 21% O_2, <0.1% O_2 (6 h) or irradiated (IR) (analyzed 1 h after 5 Gy treatment). Cells were stained for 53BP1 and DAPI, and the number of cells with >5 foci/nucleus are quantified. Bar shows the average from 3 biological repeats. The two-tailed, unpaired Student's *t*-test was used.

B. Representative images from part A. 53BP1 (green) and DAPI (blue) are shown. Scale bar represents 2.5 μM.

C. A549 cells were treated with DMSO or NAC (20 mM), exposed to 21 or <0.1% O_2 (6 h) and labeled with 5'EU (0.5 mM). The mean nuclear intensity of 5'EU per cell was quantified. DRB (100 μM, 1 h)

was used as a control. Each dot represents a nucleus, where at least 100 nuclei were imaged per condition. The black line represents average nuclear EU intensity. *n*=1

A-C Data from three independent experiments (*n*=3), mean ± standard error of the mean (SEM) are displayed unless otherwise indicated. * p <0.05, ** p <0.01, *** p <0.001, **** p <0.0001, ns (non-significant) p >0.05. Unless otherwise indicated statistical significance refers to comparison to the normoxic control. A minimum of 100 cells was imaged per condition in all microscopy experiments.

Figure S5. Linking R-loop accumulation in hypoxia with chromatin changes

A. Quantification of H3K9me2 (normalized to total H3) in western blot from Figure 6C from HCT116 cells treated with Chaetocin (1 µM) and exposed to 21 or <0.1% O_2 for 6 h.

B. Quantification of H3K9me3 (normalized to total H3) in western blot from Figure 6C from HCT116 cells treated with Chaetocin (1 µM) and exposed to 21 or <0.1% O_2 for 6 h.

C. Quantification of H3K9me3 (normalized to total H3) from Figure 6D from A549 cells transfected with mock (control) or RNase $H1^{WT}$ and exposed to 21 or <0.1% O_2 (6 h).

D. Quantification of H3K9me2 (normalized to total H3) in western blot from Figure 6F from HCT116 cells treated with NAC (20 mM) and exposed to 21 or <0.1% O_2 for 6 h.

E. Quantification of H3K9me3 (normalized to total H3) in western blot from Figure 6F from HCT116 cells treated with NAC (20 mM) and exposed to 21 or <0.1% O_2 for 6 h.

F. A549 cells were transfected with V5-tagged RNase $H1^{D210N}$ and treated with Chaetocin (1 µM) or UNC0638 (3 µM, pretreatment of 16 hours) and exposed to 6 h of 21 or <0.1% O_2. Cells were fixed and stained for V5 and the nuclear intensity was determined. CPT (10 µM, 1 h) and DRB (100 µM, 1 h) were used as controls to increase and decrease R-loop levels, respectively. Each data point represents a field of view of cells and at least 100 cells were quantified per condition. *n*=1

A-F Data from three independent experiments (*n*=3), mean ± standard error of the mean (SEM) are displayed unless otherwise indicated. * p <0.05, ** p <0.01, *** p <0.001, **** p <0.0001, ns (non-

significant) $p > 0.05$. Unless otherwise indicated statistical significance refers to comparison to the normoxic control. In parts **A-E**, each data point represents the average from one of three biological repeats, normalized to the untreated sample. The two-tailed, unpaired Student's *t*-test was used in parts **A-E.**

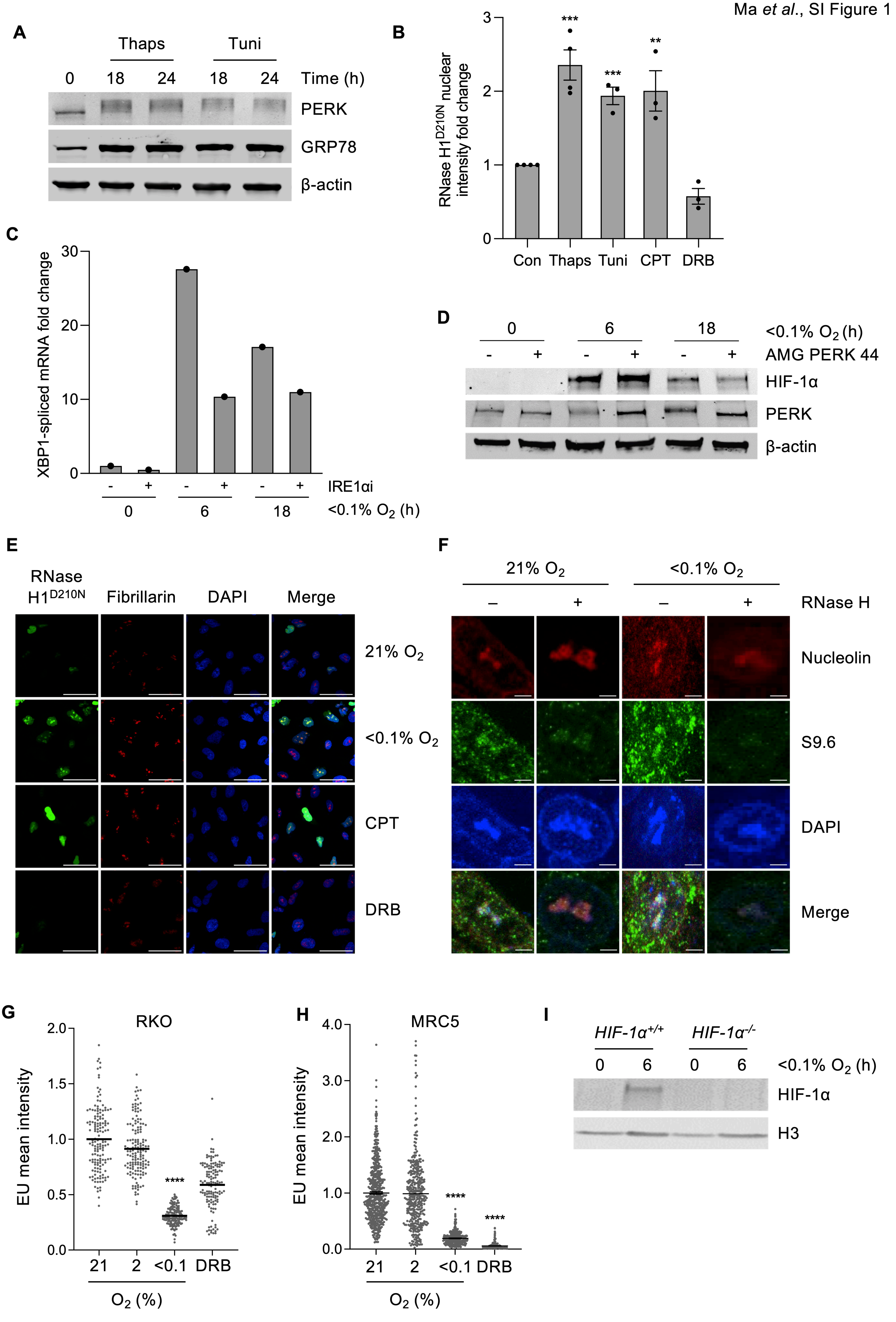
A
Thaps
Tuni
0
18
24
18
24
Time (h)
PERK
GRP78
β-actin
B
RNase H1D210N nuclear intensity fold change
***
***
**
Con
Thaps
Tuni
CPT
DRB
C
XBP1-spliced mRNA fold change
IRE1αi
<0.1% O2 (h)
D
<0.1% O2 (h)
AMG PERK 44
HIF-1α
PERK
β-actin
E
RNase H1D210N
Fibrillarin
DAPI
Merge
21% O2
<0.1% O2
CPT
DRB
F
21% O2
<0.1% O2
RNase H
Nucleolin
S9.6
DAPI
Merge
G
RKO
EU mean intensity
****
21
2
<0.1
DRB
O2 (%)
H
MRC5
EU mean intensity
****
****
21
2
<0.1
DRB
O2 (%)
I
HIF-1α+/+
HIF-1α-/-
<0.1% O2 (h)
HIF-1α
H3

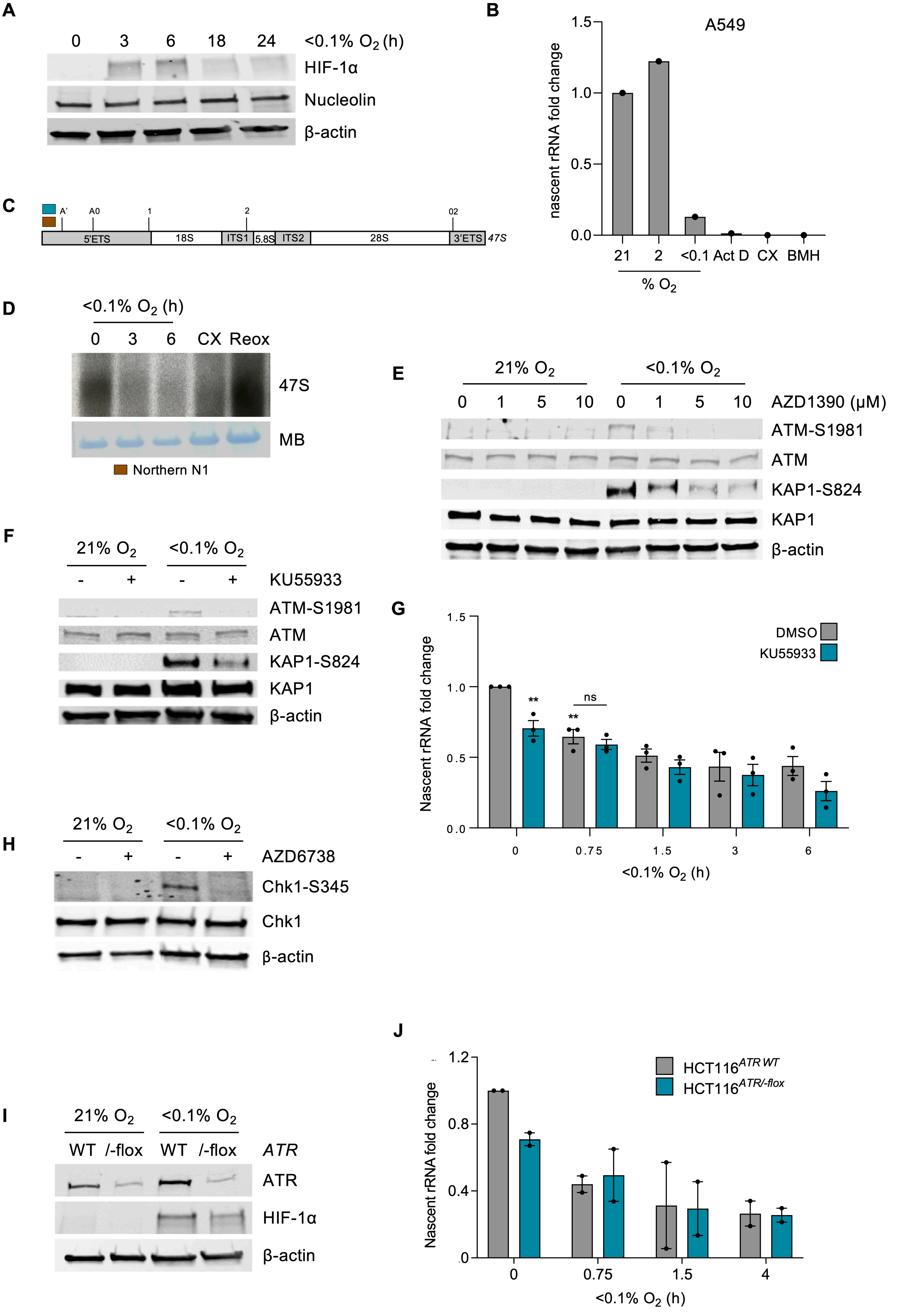
A
0
3
6
18
24
<0.1% O2 (h)
HIF-1α
Nucleolin
β-actin
B
A549
nascent rRNA fold change
1.5
1.0
0.5
0.0
21
2
<0.1
Act D
CX
BMH
% O2
C
A'
A0
1
2
02
5'ETS
18S
ITS1
5.8S
ITS2
28S
3'ETS
47S
D
<0.1% O2 (h)
0
3
6
CX
Reox
47S
MB
Northern N1
E
21% O2
<0.1% O2
0
1
5
10
AZD1390 (μM)
ATM-S1981
ATM
KAP1-S824
KAP1
β-actin
F
21% O2
<0.1% O2
KU55933
ATM-S1981
ATM
KAP1-S824
KAP1
β-actin
G
Nascent rRNA fold change
DMSO
KU55933
**
ns
0.75
1.5
3
6
<0.1% O2 (h)
H
21% O2
<0.1% O2
AZD6738
Chk1-S345
Chk1
β-actin
I
21% O2
<0.1% O2
WT
/-flox
ATR
ATR
HIF-1α
β-actin
J
Nascent rRNA fold change
1.2
0.8
0.4
.0
HCT116ATR WT
HCT116ATR/-flox
0
0.75
1.5
4
<0.1% O2 (h)

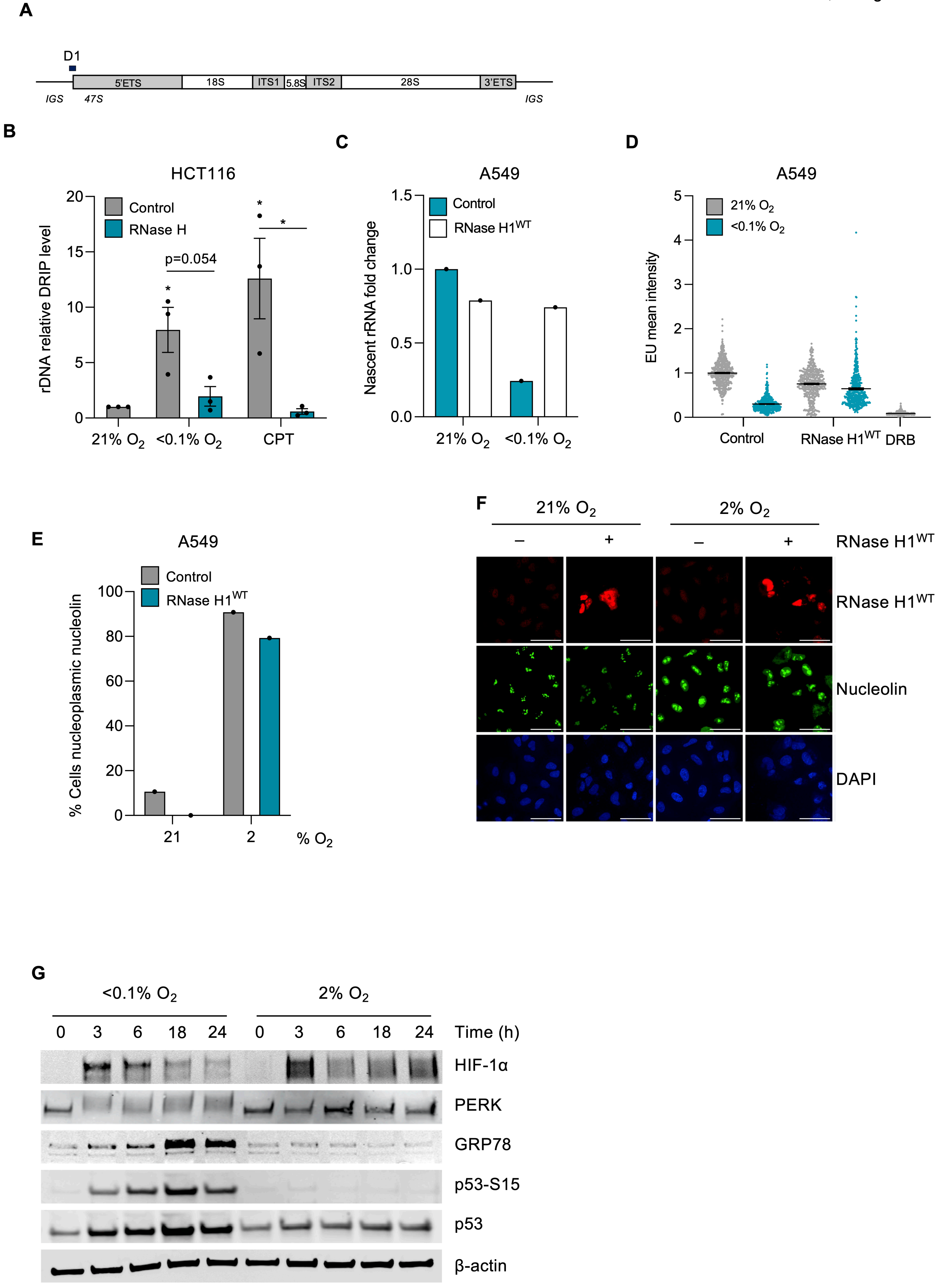
A
D1
5'ETS
18S
ITS1
5.8S
ITS2
28S
3'ETS
IGS
47S
IGS
B
HCT116
rDNA relative DRIP level
Control
RNase H
p=0.054
21% O2
<0.1% O2
CPT
C
A549
Nascent rRNA fold change
Control
RNase H1WT
21% O2
<0.1% O2
D
A549
EU mean intensity
21% O2
<0.1% O2
Control
RNase H1WT
DRB
E
A549
% Cells nucleoplasmic nucleolin
Control
RNase H1WT
21
2
% O2
F
21% O2
2% O2
RNase H1WT
RNase H1WT
Nucleolin
DAPI
G
<0.1% O2
2% O2
0 3 6 18 24 0 3 6 18 24
Time (h)
HIF-1α
PERK
GRP78
p53-S15
p53
β-actin

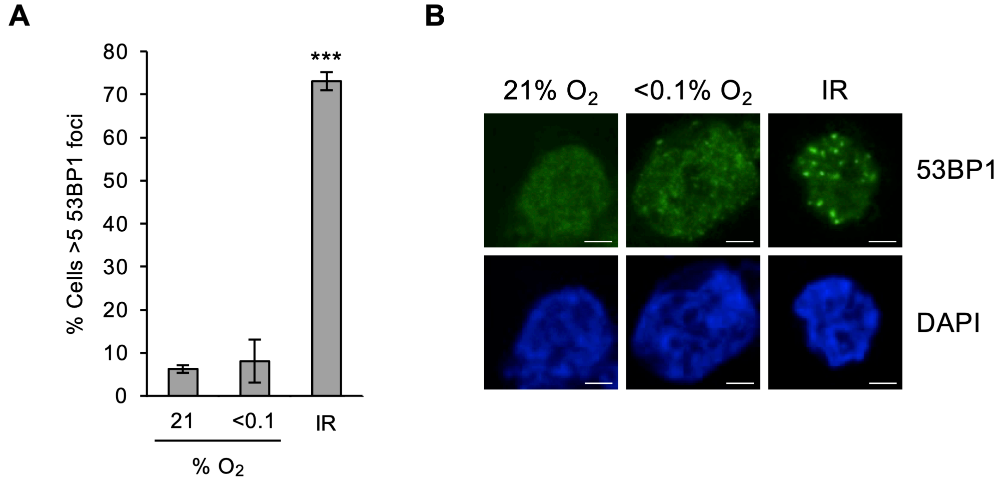
A
% Cells >5 53BP1 foci
80
70
60
50
40
30
20
10
0
***
21
<0.1
IR
% O2
B
21% O2
<0.1% O2
IR
53BP1
DAPI

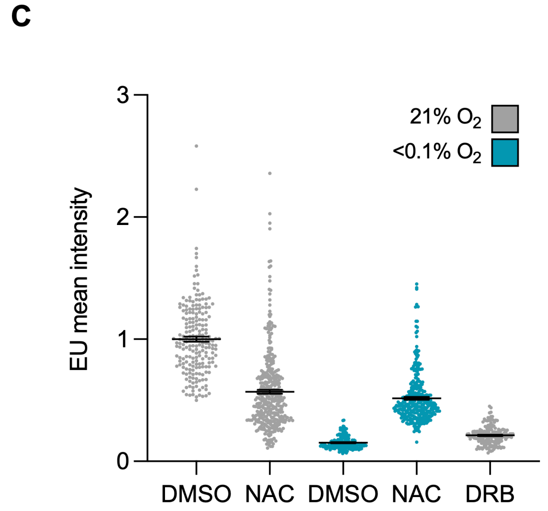
C
EU mean intensity
3
2
1
0
21% O2
<0.1% O2
DMSO
NAC
DMSO
NAC
DRB

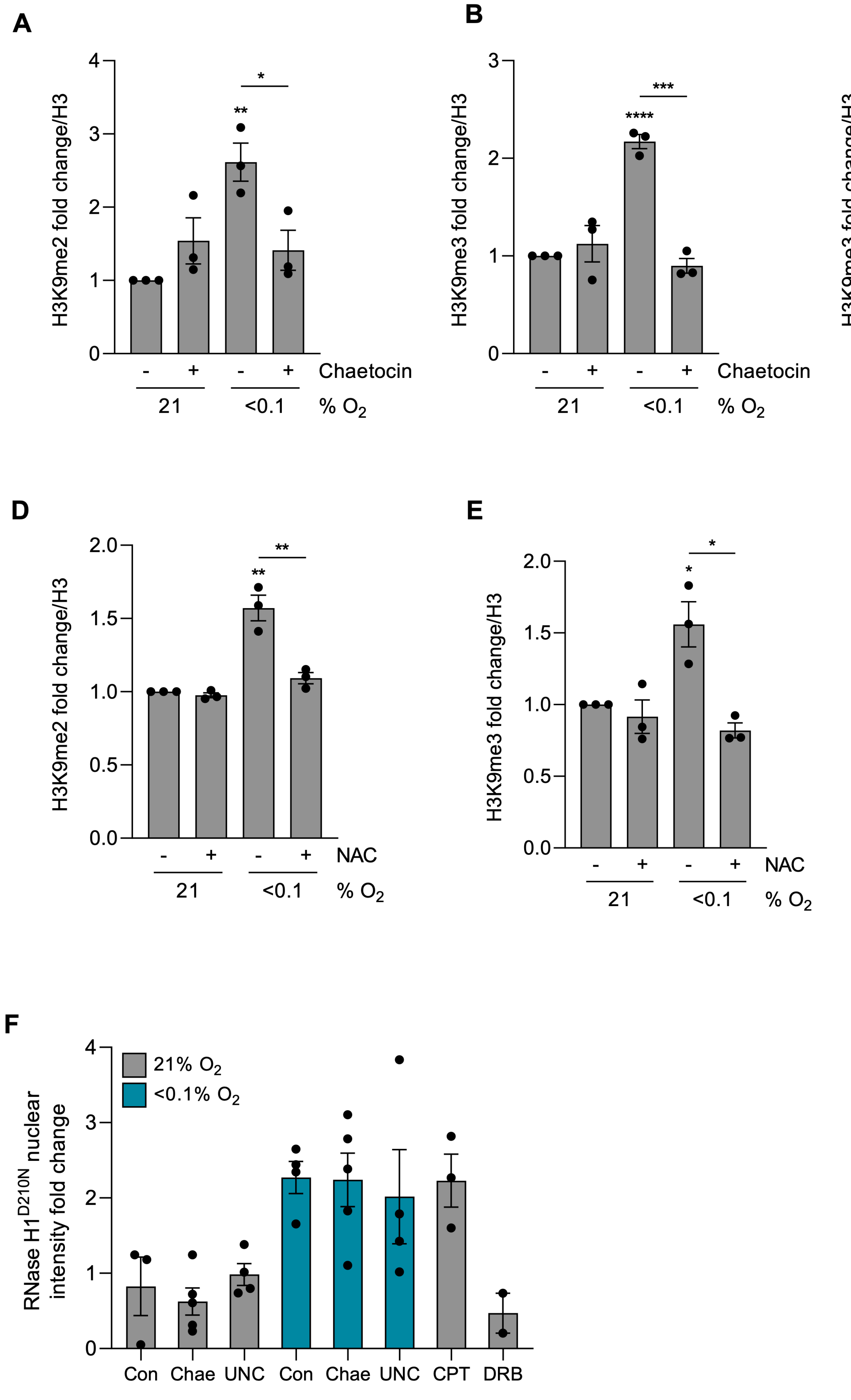
A
H3K9me2 fold change/H3
*
**
Chaetocin
% O2
21
<0.1
B
H3K9me3 fold change/H3
***
****
Chaetocin
% O2
21
<0.1
C
H3K9me3 fold change/H3
ns
***
RNase H1WT
% O2
21
<0.1
D
H3K9me2 fold change/H3
**
**
NAC
% O2
21
<0.1
E
H3K9me3 fold change/H3
*
*
NAC
% O2
21
<0.1
F
RNase H1D210N nuclear intensity fold change
21% O2
<0.1% O2
Con
Chae
UNC
Con
Chae
UNC
CPT
DRB
